# Synergistic Antifungal Effect of a Combination of Iron Deficiency and Calcium Supplementation

**DOI:** 10.1128/spectrum.01121-22

**Published:** 2022-06-08

**Authors:** Jing Ye, Yamei Wang, Xinyu Li, Qinyi Wan, Yuanwei Zhang, Ling Lu

**Affiliations:** a Jiangsu Key Laboratory for Microbes and Functional Genomics, Jiangsu Engineering and Technology Research Centre for Microbiology, College of Life Sciences, Nanjing Normal University, Nanjing, China; Universidade de Sao Paulo

**Keywords:** *Aspergillus fumigatus*, antifungal therapy, calcium, iron

## Abstract

Fungal diseases have become a major public health issue worldwide. Increasing drug resistance and the limited number of available antifungals result in high morbidity and mortality. Metal-based drugs have been reported to be therapeutic agents against major protozoan diseases, but knowledge of their ability to function as antifungals is limited. In this study, we found that calcium supplementation combined with iron deficiency causes dramatic growth inhibition of the human fungal pathogens Aspergillus fumigatus, Candida albicans, and Cryptococcus neoformans. Calcium induces the downregulation of iron uptake-related genes and, in particular, causes a decrease in the expression of the transcription factor HapX, which tends to transcriptionally activate siderophore-mediated iron acquisition under iron-deficient conditions. Iron deficiency causes calcium overload and the overproduction of intracellular reactive oxygen species (ROS), and perturbed ion homeostasis suppresses fungal growth. These phenomena are consistently identified in azole-resistant A. fumigatus isolates. The findings here imply that low iron availability lets cells mistakenly absorb calcium as a substitute, causing calcium abnormalities. Thus, there is a mutual effect between iron and calcium in fungal pathogens, and the combination of calcium with an iron chelator could serve to improve antifungal therapy.

**IMPORTANCE** Millions of immunocompromised people are at a higher risk of developing different types of severe fungal diseases. The limited number of antifungals and the emergence of antimicrobial resistance highlight an urgent need for new strategies against invasive fungal infections. Here, we report that calcium can interfere with iron absorption of fungal pathogens, especially in iron-limited environments. Thus, a combination of calcium supplementation with an iron chelator inhibits the growth of human fungal pathogens, including Aspergillus fumigatus, Candida albicans, and Cryptococcus neoformans. Moreover, we demonstrate that iron deficiency induces a nonspecific calcium uptake response, which results in toxic levels of metal. Findings in this study suggest that a microenvironment with excess calcium and limited iron is an efficient strategy to curb the growth of fungal pathogens, especially for drug-resistant isolates.

## INTRODUCTION

Metals are essential nutrients for all forms of life and are required for the functions of nearly half of all enzymes that are involved in all fundamental biological processes (1). When metals are present in growth medium at low concentrations, in many cases, they mainly serve as enzymatic cofactors and mediate electron transport (2–4). Accordingly, a lack of or a deficiency in the ability of eukaryotic cells to obtain these essential metals could result in cell sickness or death (1, 5). In contrast, when the required metal concentrations are exceeded, the metals become toxic to the cell (6). Based on the biological characterizations of metals, mammalian hosts have evolved sophisticated defense mechanisms by competing for limited metals or sequestering some metals in macrophages to kill phagocytic pathogens (7). Therefore, metal ions are also involved in the nutritional immunity by which the hosts protect themselves against invading pathogens (8). Correspondingly, the abilities of opportunistic pathogens to obtain metal ions from environmental nutrients during the process of infection could determine virulence and could therefore be used as potential new drug targets (9). Therefore, from the ancient history of medicine to the pioneering times of modern pharmacology, metal-based drugs have been used as therapeutic agents, especially as anti-infective agents against the major protozoan diseases (10–12).

Invasive fungal infections, mainly caused by *Candida*, Aspergillus, and Cryptococcus spp., are seemingly increasing worldwide, especially in immunocompromised patients (13–15). Aspergillus fumigatus is a notorious pathogen responsible for invasive aspergillosis (16, 17). As a saprophytic filamentous fungus, A. fumigatus is a successful human pathogen due to the unique biological characteristics of the saprophytic lifestyle, and the selective pressure encountered in its primary ecological niche contributes to its virulence, including swift adaptability to the host environment, which enables the fungal cells to survive in harsh environments and evade host immune surveillance (18). Moreover, the steady increase in drug resistance of fungal pathogens reduces the efficacy of the existing antifungals (19), and new target-specific antifungal drugs are difficult to find, since both the fungal and host cells are eukaryotic. Therefore, the identification and characterization of fungal virulence factors as potential new drug targets is very important, especially for treating infections caused by existing drug-resistant pathogens.

Iron is an essential micronutrient that participates as an active redox cofactor in many enzymes and electron transfer proteins that are required for central cellular processes (20, 21). For most pathogens, including A. fumigatus, iron uptake is a critical virulence factor (22, 23). In fact, pathogens have evolved sophisticated systems to acquire iron from the host to overcome the low iron bioavailability during invasion (1, 20, 24, 25). A. fumigatus has a virulence-required siderophore-mediated iron acquisition system (26). The major secreted siderophore is triacetylfusarinine C (TAFC) (27, 28), which is inducibly secreted under iron-deficient conditions. In A. fumigatus, iron uptake, consumption and detoxification must be tightly controlled. To maintain iron homeostasis, A. fumigatus has evolved two major reverse-regulated (29) transcription factors, SreA and HapX (30–32), which function under iron-sufficient and iron-deficient conditions, respectively. Therefore, fungal pathogens have developed sophisticated systems to control the uptake, expansion, and storage for iron homeostasis, which are critical for various physiological processes.

Calcium, a key second messenger and the most abundant metal in the human body, plays a vital role in the physiology of organisms (33, 34). Similar to other metal uptake mechanisms, low-affinity (FigA) and high-affinity (CchA and MidA) calcium uptake systems in A. fumigatus have been described (35–37). These mutants are more sensitive to reactive oxygen species (ROS), and the mutations are also related to antifungal susceptibility and attenuated virulence (37). The intracellular Ca^2+^ concentration is strictly and precisely controlled by a sophisticated calcium homeostasis system, which is composed of various calcium channels, calcium pumps, and calcium antiporters (38–40). In fungi, mitochondria and vacuoles serve as the major Ca^2+^ stores and play critical roles in the detoxification of cytoplasmic Ca^2+^ (39, 41–43). The mitochondria in A. fumigatus have been found to store large amounts of Ca^2+^ via uptake through the Ca^2+^ uniporter McuA, located in the mitochondrial membrane (42), which plays a central role in cell physiology by shaping cytosolic Ca^2+^ transients and regulating cell survival or death. Mitochondrial Ca^2+^ sequestration causes an increase in the production of mitochondrial ROS (44). In summary, both iron and calcium are essential but can be toxic to cells when present in excess. Notably, there have been several reports regarding the effects of calcium consumption on iron absorption in mammals. Concurrent ingestion of calcium and iron from the same meal inhibits iron absorption (45–48). This evidence implies that iron and calcium nutrients may have mutual effects in cells such that one metal deficiency may induce another nonspecific metal uptake response, which results in toxic levels of metals. However, there is limited knowledge about the relationship between iron and calcium nutrients in hosts and fungal pathogens.

In the present study, we demonstrate the mutual effect and molecular mechanism of calcium supplementation while limiting iron in three invasive human fungal pathogens: A. fumigatus, Candida albicans, and Cryptococcus neoformans.

## RESULTS

### A. fumigatus shows sensitivity to calcium under iron-deficient conditions.

**FIG 1:**
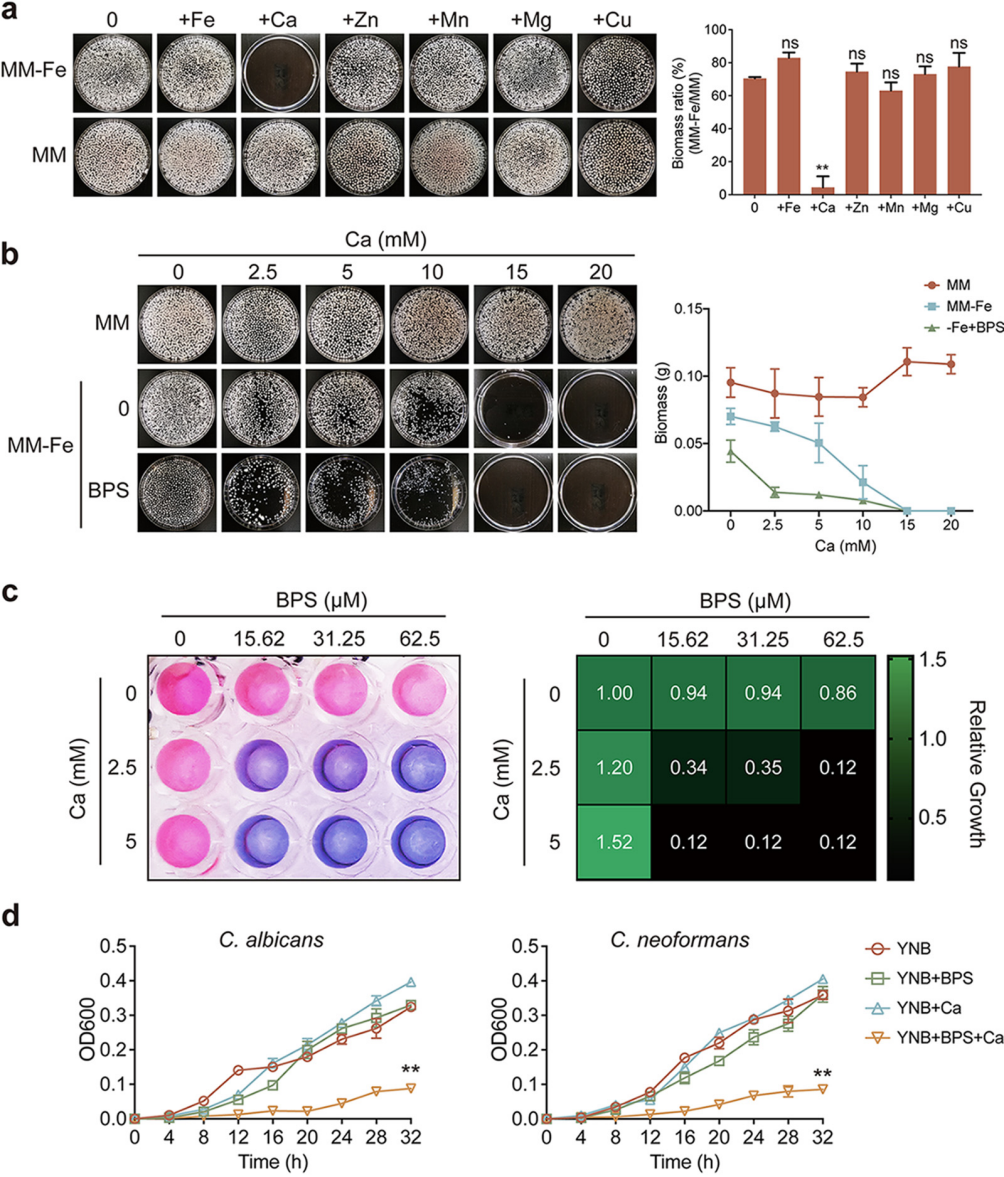
Calcium affects fungal growth under iron-deficient conditions. (a) Growth phenotypes of Aspergillus fumigatus grown in liquid MM and MM−Fe supplemented with different divalent ions, including 10 μM iron, 20 mM calcium, 100 μM zinc, 100 μM manganese, 20 mM magnesium, and 50 μM copper. Conidia (1 × 10^7^) inoculated in 100 mL of medium were scored after 24 h of growth at 37°C, and the mycelial pellets were collected on the plate with sterile water to take photos. Quantitative analysis of biomass of WT in MM and MM−Fe medium with various divalent ions. Aspergillus fumigatus was significantly inhibited under iron depletion with calcium treatment. Statistical significance was determined using a two-tailed *t* test. ns, not significant (*P* > 0.05); **, *P* < 0.01. (b) Growth in liquid media reflecting iron-replete (MM), iron-deficient (MM−Fe) and harsh iron-deficient (MM−Fe+BPS) conditions with different concentrations of calcium. Growth ability was significantly reduced in 15 mM calcium in MM−Fe, and 2.5 mM calcium inhibited growth in MM−Fe+BPS. (c) Conidia (2 × 10^3^) of the wild-type Aspergillus fumigatus were inoculated into 100 μL of MM−Fe with different concentrations of BPS in each column, supplemented with different concentrations of calcium in each row, and incubated at 37°C. After 36 h, the medium in each well was replaced with 100 μL of medium supplemented with a final concentration of 0.002% (wt/vol) resazurin. The plate was then incubated for another 4 h at 37°C. Each well represents the mean of three replicate experiments. The heat map shows the growth ability of the wild-type in different concentrations of calcium under iron-deficient conditions. Assays were performed in triplicate, and the optical density readings of fungal growth were standardized to the no-calcium-BPS control wells and are shown as relative growth values. (d) Growth curves of C. albicans and C. neoformans under iron-deficient conditions supplemented with calcium. C. albicans and C. neoformans cells (1 × 10^5^) were inoculated in liquid YNB supplemented with BPS and calcium and incubated at 37°C. Concentrations used were 20 μM BPS and 100 mM calcium for C. albicans and 30 μM BPS and 120 mM calcium for C. neoformans. The optical density (600 nm) was measured every 4 h. Statistical significance was determined using a two-way analysis of variance (ANOVA). **, *P* < 0.01.

To explore whether common niche-available divalent metals could affect acquisition of iron, which is a critical fungal growth factor (21), we measured the hyphal growth of the A. fumigatus wild-type strain in liquid iron-replete medium (minimal medium [MM]) and iron-deficient medium (MM−Fe) supplemented with various divalent ions. As shown in Fig. 1a, the growth of A. fumigatus was inhibited under iron-deficient conditions (MM−Fe); however, the addition of iron could rescue the growth defects, while supplementation with other divalent ions could not. Surprisingly, the addition of calcium exacerbated the inhibition of A. fumigatus growth under iron-deficient conditions, and these results indicated that exogenous calcium inhibited A. fumigatus growth under iron-deficient conditions. To further confirm whether this inhibition is specific to calcium, we tested the colony growth of A. fumigatus on solid media with a series of concentrations of various divalent ions (49–51). Calcium, magnesium, and copper (to some extent), but not other tested divalent ions, showed aggravated inhibition of colony growth under iron-deficient (−Fe+BPS [bathophenanthroline disulfonic acid]) conditions (see Fig. S1 in the supplemental material). These results suggested that calcium had the strongest inhibition of the wild-type strain under iron-deficient conditions.

Furthermore, we examined the biomass of A. fumigatus hyphae under iron-deficient conditions with a series of concentration gradients of calcium. As shown in Fig. 1b, hyphal growth was notably inhibited in the presence of 10 mM calcium in iron-deficient medium (MM−Fe). This inhibition was aggravated when harsh iron-deficient conditions were achieved by adding the iron chelator BPS; here, 2.5 mM calcium was sufficient to significantly inhibit growth. We further assessed the metabolic activity of A. fumigatus in the presence of calcium under iron-deficient conditions, as revealed by the color change in the resazurin assay (Fig. 1c). A blue color represents the absence of metabolic activity, and a pink color represents high metabolic activity. Consistent with the hyphal biomass assay, the combination of calcium and an iron chelator significantly inhibited the metabolic activity of A. fumigatus. Next, we were curious whether these conditions could restrict the growth of other important fungal pathogens, such as C. albicans and C. neoformans. To test this hypothesis, we examined the effects of iron-deficient conditions and calcium supplementation on the growth of wild-type C. albicans and C. neoformans strains cultured in liquid media. As shown in Fig. 1d, the growth of C. albicans and C. neoformans showed sensitivity to calcium under iron-deficient conditions, which was consistent with the findings in A. fumigatus. These results suggested that this is a universal phenomenon among fungal pathogens under *in vitro* laboratory conditions.

### The combination of a low-iron diet with calcium supplementation leads to reduced fungal fitness in an *in vivo* murine model.

**FIG 2:**
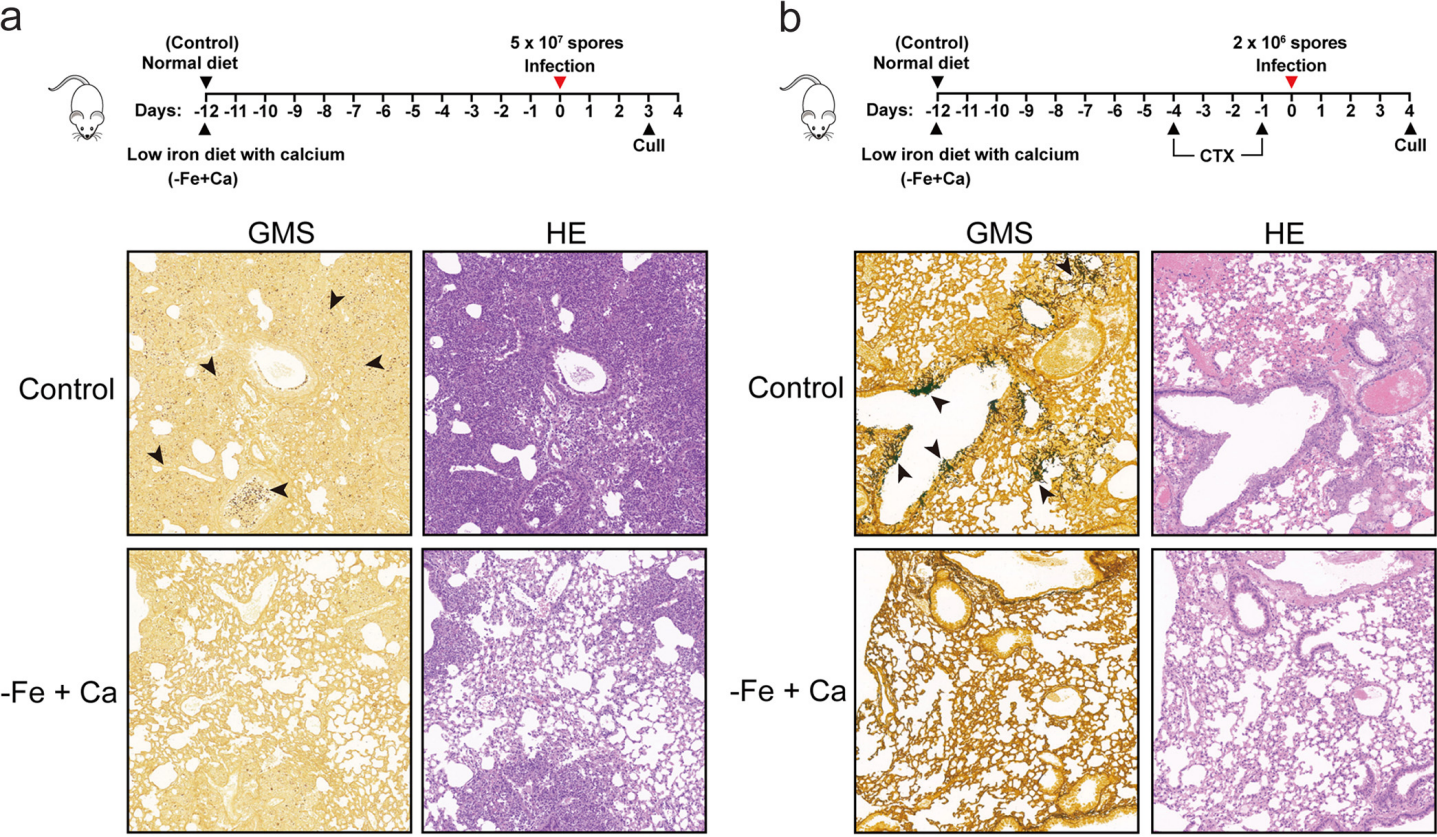
Effects of a low-iron diet with calcium supplementation on the fitness of A. fumigatus in a murine infection model. (a) Timeline of the experimental protocol establishing a normal diet (45 ppm iron) and a low-iron diet (3 ppm iron) injected with calcium (3% CaCl_2_) in 8-week-old mice. Histopathology of representative lung sections of mice fed on a normal diet and a low-iron diet with calcium supplementation at 48 h after infection with the wild-type strain. Lung sections were stained with HE for visualization of the host cells and Gomori methenamine silver (GMS) for visualization of the fungal elements. (b) Timeline of the experimental protocol establishing a normal diet (45 ppm iron) and a low-iron diet (3 ppm iron) injected with calcium (3% CaCl_2_) in 8-week-old mice, showing the time points at which cyclophosphamide (CTX) was injected. Histopathology of representative lung sections from mice receiving a normal diet and a low-iron diet with calcium supplementation at 4 days after infection with the wild-type strain. Lung sections were stained with HE for visualization of the host cells and GMS for visualization of the fungal elements.

We next wondered whether this phenomenon might occur in an *in vivo* murine model. A murine model of iron deficiency with calcium supplementation was established in 8-week-old mice on a low (3-ppm)-iron diet and receiving intragastric injection with calcium for 12 consecutive days (Fig. 2a) (52–54). Then, the immunocompetent mice were directly challenged with 5 × 10^7^ conidia of the wild-type strain via the trachea. After 2 days of infection, the group on the low-iron diet with calcium supplementation displayed a decrease in lung tissue damage, as determined by hematoxylin-and-eosin (HE) staining and silver staining of lung tissue sections (Fig. 2a). In comparison, the control group fed a normal diet showed severe lesions located mainly around the bronchi, and considerable A. fumigatus hyphae were also seen. Moreover, using the neutropenic murine lung infection model, we observed few fungal hyphae in mice receiving the low-iron diet with calcium supplement, while control group infections presented multiple foci of invasive hyphal growth (Fig. 2b). Together, these results suggested that the combination of a low-iron diet with calcium supplementation resulted in reduced fungal fitness in an *in vivo* murine model.

### A. fumigatus iron uptake-defective mutants show increased sensitivity to calcium.

**FIG 3:**
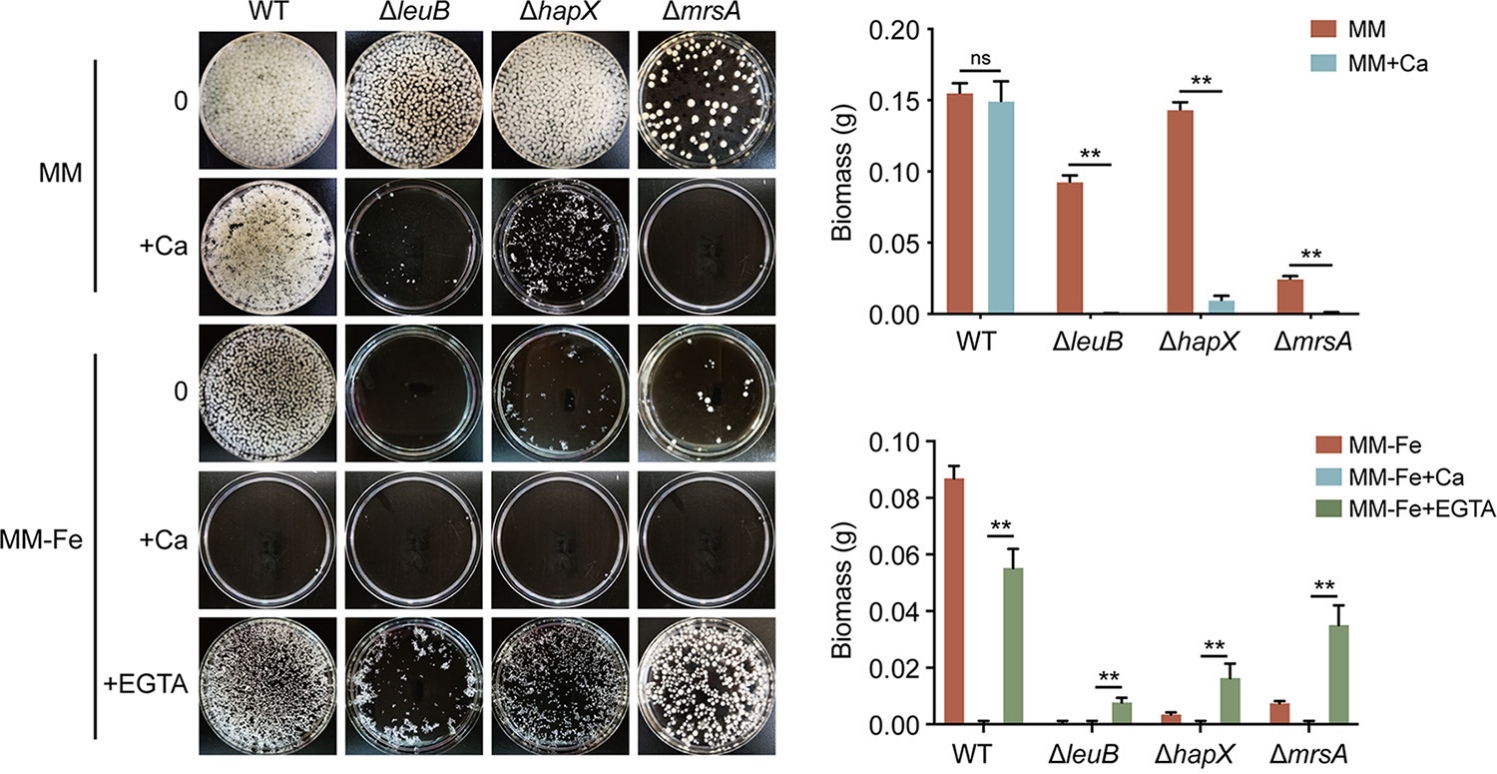
A. fumigatus iron-deficient mutants show increased sensitivity to calcium. Growth phenotypes of the wild-type 1160^c^ (WT), Δ*leuB*, Δ*hapX*, and Δ*mrsA* strains grown in liquid MM and MM−Fe supplemented with calcium. EGTA was added to MM−Fe to test the growth ability in liquid media. Biomass production of 10^7^ conidia inoculated into 100 mL of media was scored after 24 h of growth at 37°C and normalized to that of the WT in MM (the Δ*mrsA* strain was grown for 36 h). Statistical significance was determined using a two-way ANOVA. ns, not significant (*P* > 0.05); **, *P* < 0.01.

The above-mentioned results showed that calcium inhibited fungal growth under exogenous iron-deficient conditions *in vivo* and *in vitro*. We next questioned whether iron uptake-defective mutants displayed sensitivity to calcium. Thus, we inoculated the conidia of the mutants that were previously shown to have defective iron uptake in liquid iron-deficient medium, including null mutants of the transcription factors involved in iron acquisition, *leuB* and *hapX*, and the putative mitochondrial iron transporter *mrsA* (31, 55, 56), into liquid MM supplemented with calcium. As shown in Fig. 3, after 2 days of culture, the iron uptake-defective Δ*leuB*, Δ*hapX*, and Δ*mrsA* mutants displayed increased sensitivity to calcium in iron-replete MM, and these defects became more pronounced under iron-deficient conditions (MM−Fe) compared with the wild-type strain (Fig. 3). Furthermore, we explored the calcium sensitivity for the *sreA* deletion mutant, which displays derepressed iron uptake, leading to increased cellular iron accumulation (32). As shown in Fig. S2, in contrast to A. fumigatus iron uptake-defective mutants, the mutant with the deletion of *sreA* showed a slightly increased resistance to calcium on harsh iron-deficient medium. Together, these results suggested that decreased iron acquisition is related to calcium susceptibility. Interestingly, adding the calcium chelator EGTA significantly rescued the growth defects of these mutants under iron-deficient conditions, suggesting that exogenous calcium is toxic to iron uptake-defective mutants of A. fumigatus.

### The expression of genes involved in calcium and iron homeostasis is affected under iron-deficient conditions in response to calcium stimuli.

**FIG 4:**
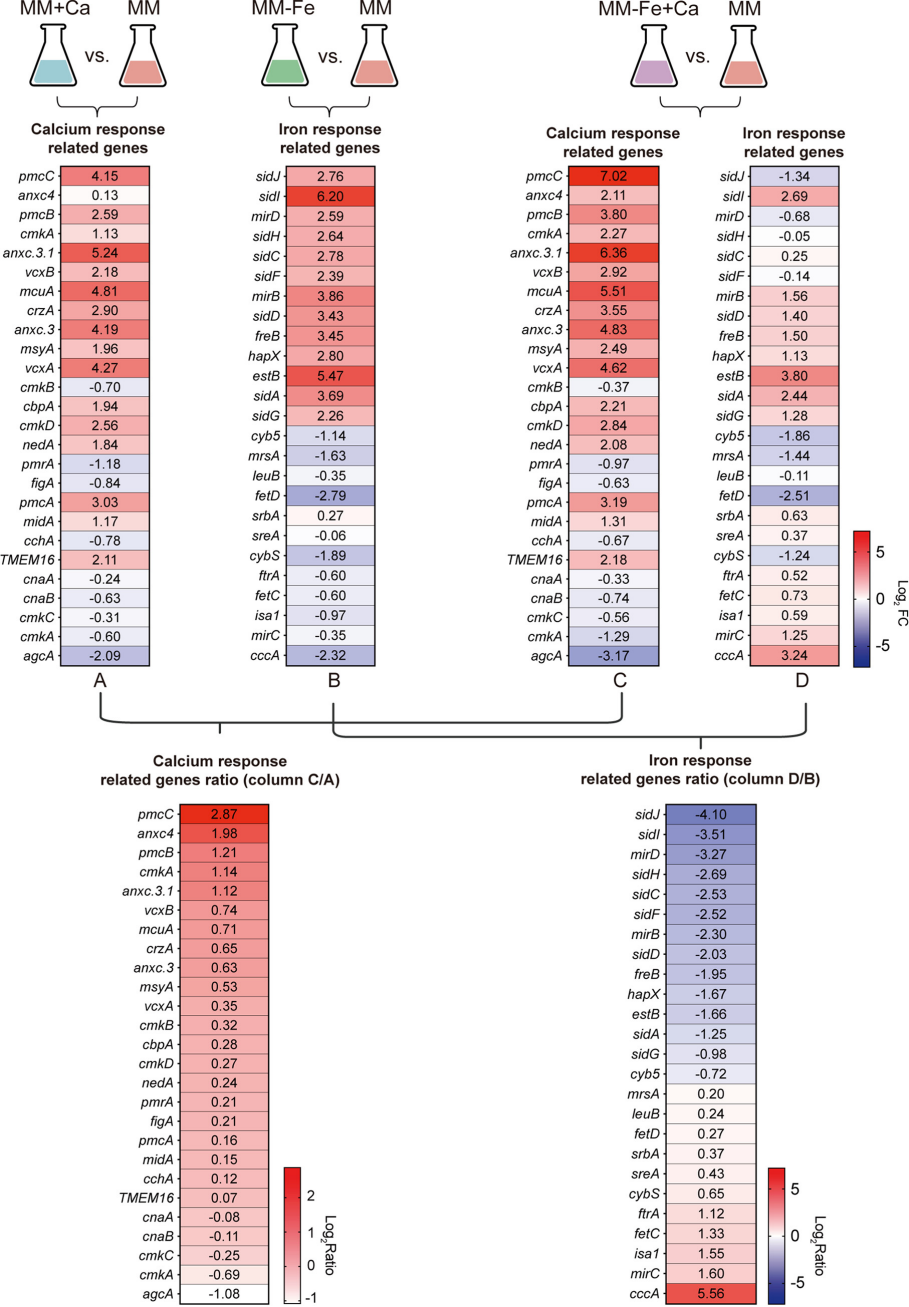
Expression of the genes involved in calcium and iron homeostasis was affected under iron-deficient conditions in response to calcium stimulus. RNA-seq analysis of the whole-genome transcript profiles of the wild-type in MM and MM−Fe in response or not to calcium stimulus. Heat maps show the comparison of the RNA-seq data after different treatments to those for the wild-type strain in MM. Column A shows the selected genes putatively involved in calcium signal transduction after 10 min of 200 mM calcium stress in MM (MM+Ca) versus time zero MM (24 h of growth). Column B shows the selected genes putatively involved in iron metabolism in MM−Fe versus MM (24 h of growth). Column C shows the selected genes putatively involved in calcium signal transduction after 10 min of 200 mM calcium stress in MM−Fe (MM−Fe+Ca) versus MM (24 h of growth). Column D shows the selected genes putatively involved in iron metabolism after 10 min of 200 mM calcium stress in MM−Fe (MM−Fe+Ca) versus MM (24 h of growth). Data are the averages and standard deviations from three independent biological repetitions.

To explore the underlying mechanism of A. fumigatus growth inhibition in the presence of calcium under iron-deficient conditions, we carried out transcriptome sequencing (RNA-seq) analysis to compare the transcriptional profiles between liquid culture MM (iron-replete) and MM−Fe (iron-deficient) after exposure to 200 mM calcium. Given that iron deficiency and calcium supplementation may affect metal ion homeostasis, we focused specifically on the genes involved in calcium and iron homeostasis. When cells were cultured in liquid minimal medium and challenged with calcium, the genes that are responsible for calcium storage, such as calcium transporters, calcium channels, and protein kinases, were upregulated (Fig. 4, column A), which is consistent with previous reports (29, 57, 58). Interestingly, under iron-deficient conditions, genes for calcium-related proteins, such as vacuolar calcium pump genes *pmcB* and *pmcC* and the mitochondrial calcium uniporter gene *mcuA*, exhibited a higher response than in minimal medium when the fungal cells were challenged with calcium (Fig. 4, column C and the column C/A ratio), implying that iron-deficient conditions could lead to the elevated activation of calcium response-related genes. Additionally, iron-deficient conditions induced the expression of genes involved in reductive iron assimilation and the siderophore biosynthetic pathway, such as *sidJ*, *sidI*, *sidH*, and *mirB* (Fig. 4, column B), as reported previously (26, 59–63). After exposure to calcium for 10 min, we observed that the exogenous calcium inhibited the activation of these genes (Fig. 4, column D and the column D/B ratio). Quantitative real-time PCR (qRT-PCR) experiments validated the RNA-seq results for the 12 selected genes (Fig. S3a and b). These results suggested that calcium supplementation inhibits the expression of iron deficiency-responsive genes. Meanwhile, the iron-deficient condition leads to the elevated expression of calcium response-related genes.

### Iron deficiency causes a significant increase in extracellular calcium influx through the calcium channels CchA and MidA.

**FIG 5:**
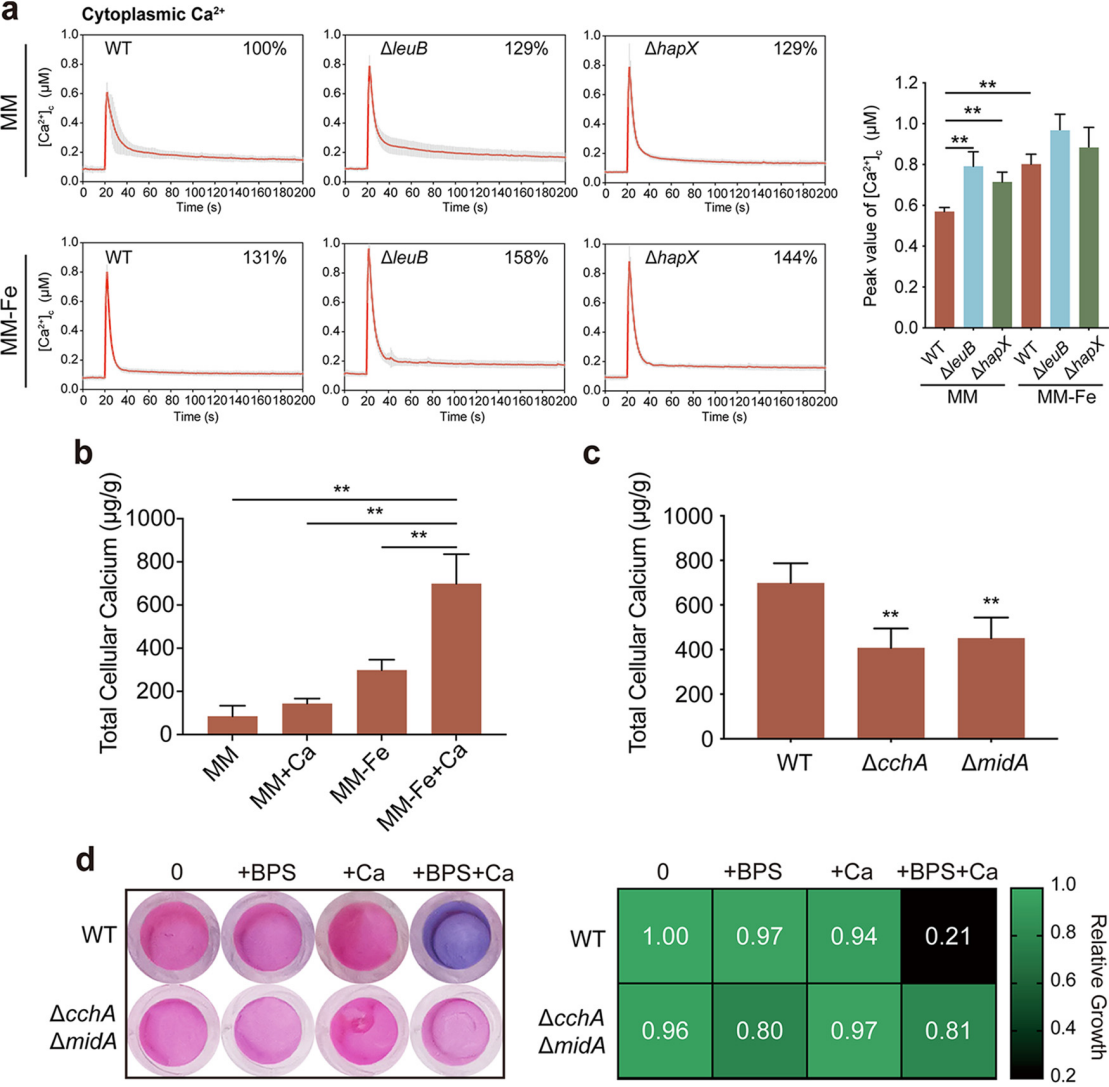
Iron deficiency causes a significant increase in extracellular calcium influx through the calcium channels CchA and MidA. (a) Cytosolic Ca^2+^ ([Ca^2+^]_c_) responses in the parental wild-type, Δ*leuB*, and Δ*hapX* strains following a high external calcium stimulus (0.1 mM CaCl_2_) under liquid iron-replete (MM) and iron-deficient (MM−Fe) conditions. The bar graph shows the peak value of the [Ca^2+^]_c_ amplitude. Statistical significance was determined using a two-tailed *t* test. **, *P* < 0.01. (b) The WT was grown for 24 h under iron-replete and iron-deficient conditions with or without 1 h of 200 mM CaCl_2_ stimulus. The calcium contents in the wild type in MM, MM+Ca, MM−Fe, and MM−Fe+Ca were measured. The results are the means from three separate experiments, and error bars show the standard deviations. Statistical significance was determined using a two-tailed *t* test. **, *P* < 0.01. (c) Calcium contents of the wild-type, Δ*cchA*, and Δ*midA* strains under iron-deficient conditions with 1 h of 200 mM CaCl_2_ stimulus. Statistical significance was determined using a two-tailed *t* test. **, *P* < 0.01. (d) Conidia (2 × 10^3^) of the Δ*cchA* Δ*midA* mutant and wild-type Aspergillus fumigatus were inoculated in 200 μL of MM−Fe with 15.625 μM BPS and 2.5 mM calcium at 37°C. After 36 h, the medium in each well was replaced with 100 μL of medium supplemented with a final concentration of 0.002% (wt/vol) resazurin. The plate was then incubated for another 4 h at 37°C. Each dot represents the mean of three replicate experiments. The heat map shows the growth ability of the Δ*cchA* Δ*midA* mutant with 2.5 mM calcium under iron-deficient conditions. Assays were performed in triplicate, the optical density readings of fungal growth were standardized to the no-calcium-BPS control wells, and the results are given as relative growth values.

Given that hyperactivation of several calcium response-related genes involved in calcium storage was observed under iron-deficient conditions, we wondered whether iron-deficient conditions could truly affect the calcium transient changes (the calcium signal) in A. fumigatus. Therefore, we constructed wild-type and iron uptake-defective mutants expressing codon-optimized aequorin. The Ca^2+^-dependent emission of blue light from aequorin was used to monitor cytosolic Ca^2+^ concentration (64). After treatment with 0.1 M CaCl_2_, transient [Ca^2+^]_c_ (the free Ca^2+^ concentration in cytosol) responses were observed, and the difference in the amplitude of [Ca^2+^]_c_ between the peak and the resting status of the parental wild-type strain was approximately 0.6 μM when the strain was grown in iron-replete medium (MM). In comparison, iron-deficient conditions (MM−Fe) resulted in a 31% rise in the [Ca^2+^]_c_ amplitude. Moreover, both the Δ*leuB* and Δ*hapX* iron uptake-defective mutants displayed increased [Ca^2+^]_c_ amplitudes (approximately 29%) compared to the parental wild-type strain under either iron-replete (MM) or iron-deficient (MM−Fe) conditions (Fig. 5a), suggesting that cytosolic calcium homeostasis was affected under iron-deficient conditions.

To further explore the underlying mechanism of iron-deficient stress-perturbed calcium homeostasis, we measured the total cellular calcium content of the wild-type strain grown in iron-replete and iron-deficient liquid cultures. As shown in Fig. 5b, the intercellular calcium content increased 2-fold under iron-deficient conditions in comparison to iron-replete conditions, suggesting that iron-deficient conditions lead to increased calcium influx. The high-affinity Ca^2+^ uptake system components CchA and MidA have been reported to be important for calcium influx in A. fumigatus (37). To examine whether the increased cellular calcium content results from calcium influx through MidA and CchA under iron-deficient conditions, we measured the total calcium contents in the Δ*cchA* and Δ*midA* calcium channel deletion mutants. The results showed that the Δ*cchA* and Δ*midA* mutants exhibited significantly decreased calcium contents compared to the wild type (Fig. 5c), suggesting that the calcium channels CchA and MidA may participate in calcium influx under iron-deficient conditions. Accordingly, the resazurin assay showed that deleting both *cchA* and *midA* rescued the growth defects in the presence of calcium under iron-deficient conditions (Fig. 5d). Taken together, these results suggest that iron deficiency causes a significant increase in extracellular calcium influx through the calcium channels CchA and MidA.

### Oxidative stress triggered by mitochondrial calcium overload contributes to the enhanced susceptibility of A. fumigatus to calcium under iron-deficient conditions.

**FIG 6:**
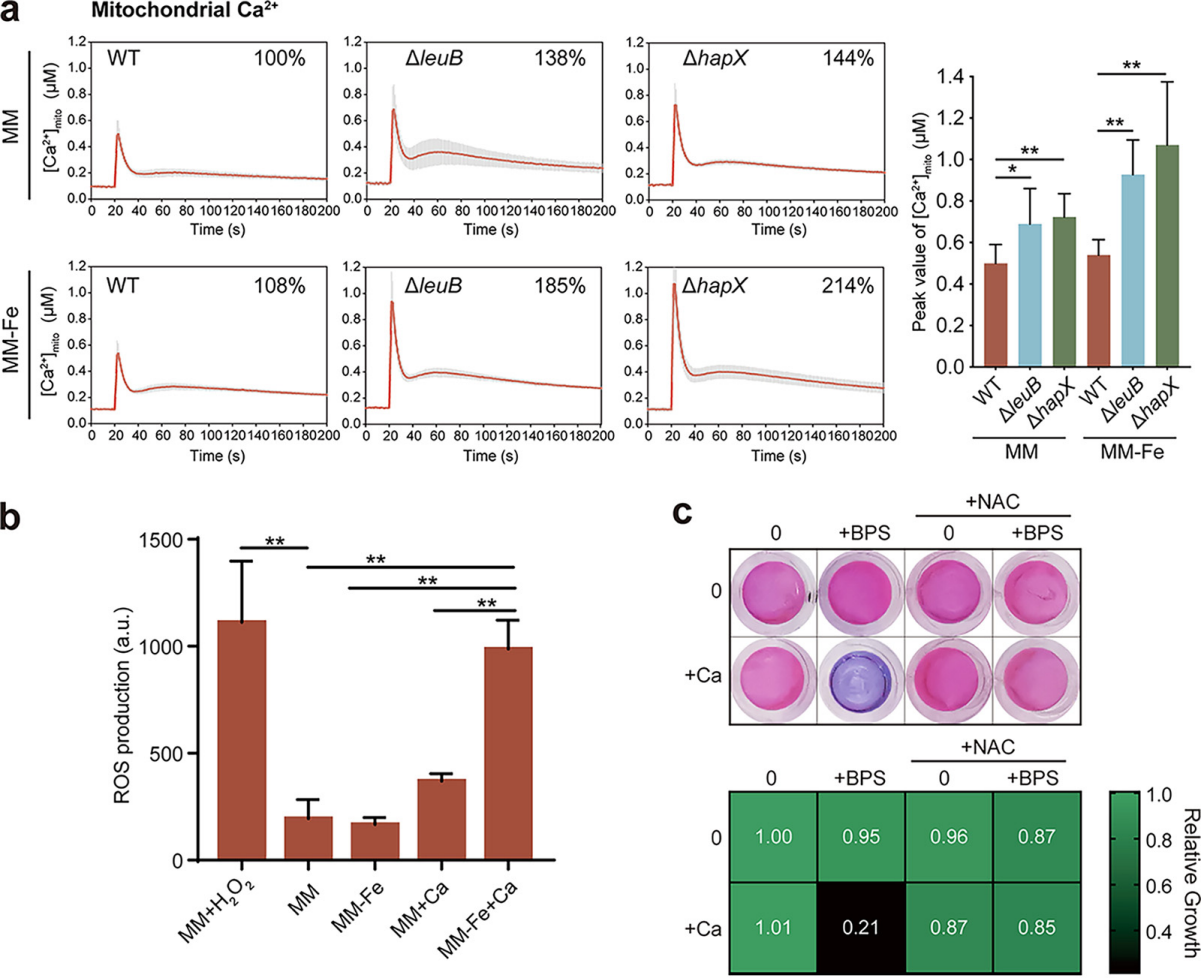
Oxidative stress triggered by mitochondrial calcium overload contributes to the enhanced susceptibility of A. fumigatus to calcium under iron-deficient conditions. (a) Mitochondrial Ca^2+^ ([Ca^2+^]_mito_) responses in the parental wild type, *leuB* mutant, and *hapX* mutant following a high external calcium stimulus (0.1 mM CaCl_2_) under liquid iron-replete (MM) and iron-deficient (MM−Fe) conditions. The bar graph shows the peak value of the [Ca^2+^]_mito_ amplitude. Statistical significance was determined using a two-tailed *t* test. *, *P* < 0.05; **, *P* < 0.01. (b) The WT was grown for 24 under iron-replete and iron-deficient conditions with or without 4 h of 200 mM CaCl_2_ stimulus. Then, 20 μM DCFH-DA was added to the medium for incubation at 37 for 1.5 h. ROS production by the wild-type in MM, MM+Ca, MM−Fe, and MM−Fe+Ca was determined. H_2_O_2_ was used as a positive control. The results are the means from three separate experiments, and error bars show the standard deviations. Statistical significance was determined using a two-tailed *t* test, **, *P* < 0.01. (c) Conidia (2 × 10^3^) of wild-type Aspergillus fumigatus were inoculated into 200 μL of MM−Fe with 15.625 μM BPS, 2.5 mM calcium, and 0.125 mM NAC at 37°C. After 36 h, the medium in each well was replaced with 100 μL of medium supplemented with a final concentration of 0.002% (wt/vol) resazurin. The plate was then incubated for another 4 h at 37°C. Each well represents the mean of three replicate experiments. The heat map shows the growth ability of the wild-type strain with calcium and NAC under iron-deficient conditions. Assays were performed in triplicate, the optical density readings of fungal growth were standardized to those of no-calcium-BPS control wells, and the results are shown as relative growth values.

To further test whether the enhanced susceptibility of A. fumigatus to calcium was related to mitochondrial calcium overload, the free Ca^2+^ concentration in the mitochondria ([Ca^2+^]_mito_) was measured by using a modified aequorin (Mt-Aeq) protein as previously reported. As shown in Fig. 6a, the mitochondrial Ca^2+^ amplitude ([Ca^2+^]_mito_) in the iron uptake-defective mutants Δ*leuB* and Δ*hapX* showed a significant increase compared to that of the wild-type strain in iron-replete MM and iron-deficient MM−Fe. These data indicated that mitochondrial calcium homeostasis may also be affected under iron-deficient conditions in A. fumigatus. It was reported that excess calcium in mitochondria generates oxidative stress in eukaryotic cells (65). Therefore, we examined the intracellular ROS levels of the wild-type using the probe 2′,7′-dichlorodihydrofluorescein diacetate (DCFH-DA), which can release fluorescence when bound to ROS; the intensity of fluorescence reflects the level of ROS production. As shown in Fig. 6b, we used H_2_O_2_-induced ROS production as a positive control. *N*-acetyl-l-cysteine ([NAC], a ROS scavenger) was used to further determine whether the increase in ROS was correlated with the growth defects of A. fumigatus in the presence of calcium under iron-deficient conditions. As shown in Fig. 6c, the addition of NAC partially rescued the observed growth defects of the wild-type strain in the presence of calcium under iron-deficient conditions, suggesting that oxidative stress triggered by mitochondrial calcium overload might contribute to the enhanced susceptibility of A. fumigatus to calcium under iron-deficient conditions.

### Exogenous calcium inhibits iron uptake by inhibiting HapX expression under iron-deficient conditions.

**FIG 7:**
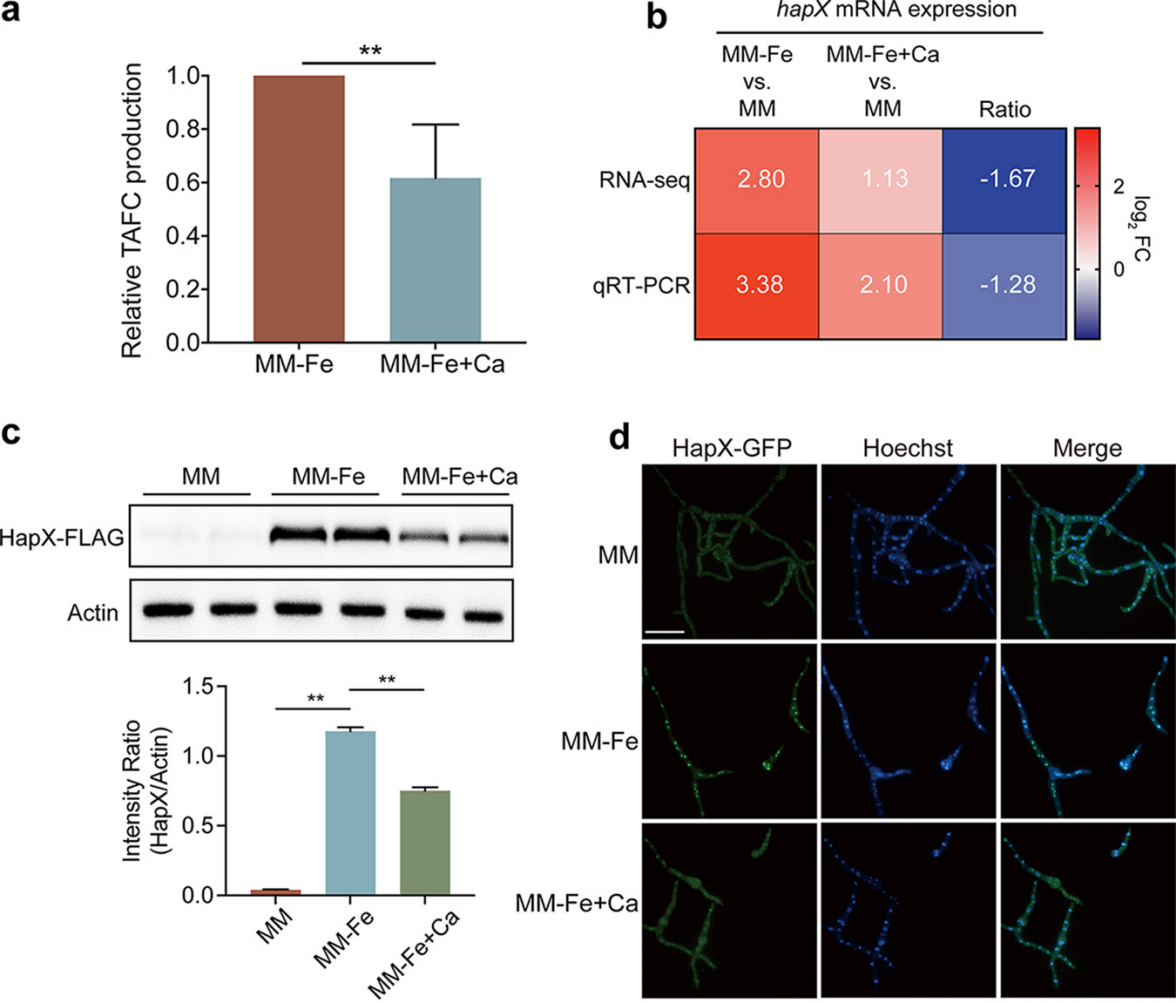
Calcium inhibits iron uptake by inhibiting HapX expression under iron-deficient conditions. (a) Quantification of the triacetylfusarinine C production of the WT after growth for 24 h at 37°C under iron-deficient conditions with and without calcium supplementation normalized to that of the WT cultured under iron-deficient conditions. Statistical significance was determined using a two-tailed *t* test. **, *P* < 0.01. (b) Heat map showing the fold change in *hapX* mRNA expression under iron-replete and iron-deficient conditions with 10 min of 200 mM calcium stimulus. The data are the means from three individually performed experiments. (c) Western blot analysis of HapX-FLAG, employing a FLAG-specific antibody, revealed dramatically reduced cleavage of FLAG under iron-deficient conditions with 30 min of 200 mM calcium stimulus compared to the WT under iron-deficient conditions. The quantified Western blot signal intensity of HapX relative to actin in the indicated strains is shown. Data are from three independent experiments. Statistical analysis was performed using two-tailed *t* tests. **, *P* < 0.01. (d) Fluorescence-microscopic images demonstrating HapX-GFP distribution with or without Ca^2+^ (200 mM) stimulation for 30 min in the wild-type strain under iron-deficient conditions. Hoechst dye was used as a nuclear localization signal to visualize the nucleus. The merged images of GFP and Hoechst staining showed nuclear localization of HapX-GFP. Bar, 10 μm.

In addition to perturbed calcium homeostasis under iron-deficient conditions, our RNA-seq data showed that the mRNA expression levels of genes involved in iron uptake, such as the siderophore biosynthesis genes *sidJ*, *sidI*, and *sidH*, were downregulated when challenged with calcium (Fig. 4); thus, we suspected that the increased calcium influx could inhibit iron uptake under iron-deficient conditions. To test this hypothesis, we compared the production of the major secreted siderophore TAFC by the wild-type after growth for 24 h under iron-deficient conditions in the presence of calcium by reversed-phase high-performance liquid chromatography (HPLC) analysis. As shown in Fig. 7a, upon calcium stimulation, TAFC production was lower than that without calcium supplementation, indicating the inhibition of TAFC production by exogenous calcium. Iron uptake and siderophore biosynthesis genes are regulated by the transcription factor HapX. Under iron-deficient conditions, HapX predominantly localizes to the nucleus, induces iron acquisition, and represses iron-consuming pathways to conserve iron. Our RNA-seq analysis found that calcium inhibits the activation of *hapX* under iron-deficient conditions, and this result was further confirmed by qRT-PCR (Fig. 7b). In line with the mRNA level, Western blot analysis showed that the protein level of HapX was significantly decreased in the presence of calcium compared to that without calcium treatment under iron-deficient conditions (Fig. 7c). Moreover, we labeled the C terminus of HapX with green fluorescent protein (GFP) under the control of its native promoter and examined its subcellular location. HapX displayed strong nuclear localization under iron-deficient conditions, whereas HapX-GFP fluorescence was barely detected in iron-replete medium. Interestingly, the addition of calcium significantly reduced the intensity of the fluorescence signals, and HapX nuclear localization was barely detected (Fig. 7d). Together, these results suggest that exogenous calcium inhibits iron uptake, probably by inhibiting HapX expression.

### Supplementation with calcium and iron chelators increases antifungal drug efficacy against azole-resistant A. fumigatus isolates.

**FIG 8:**
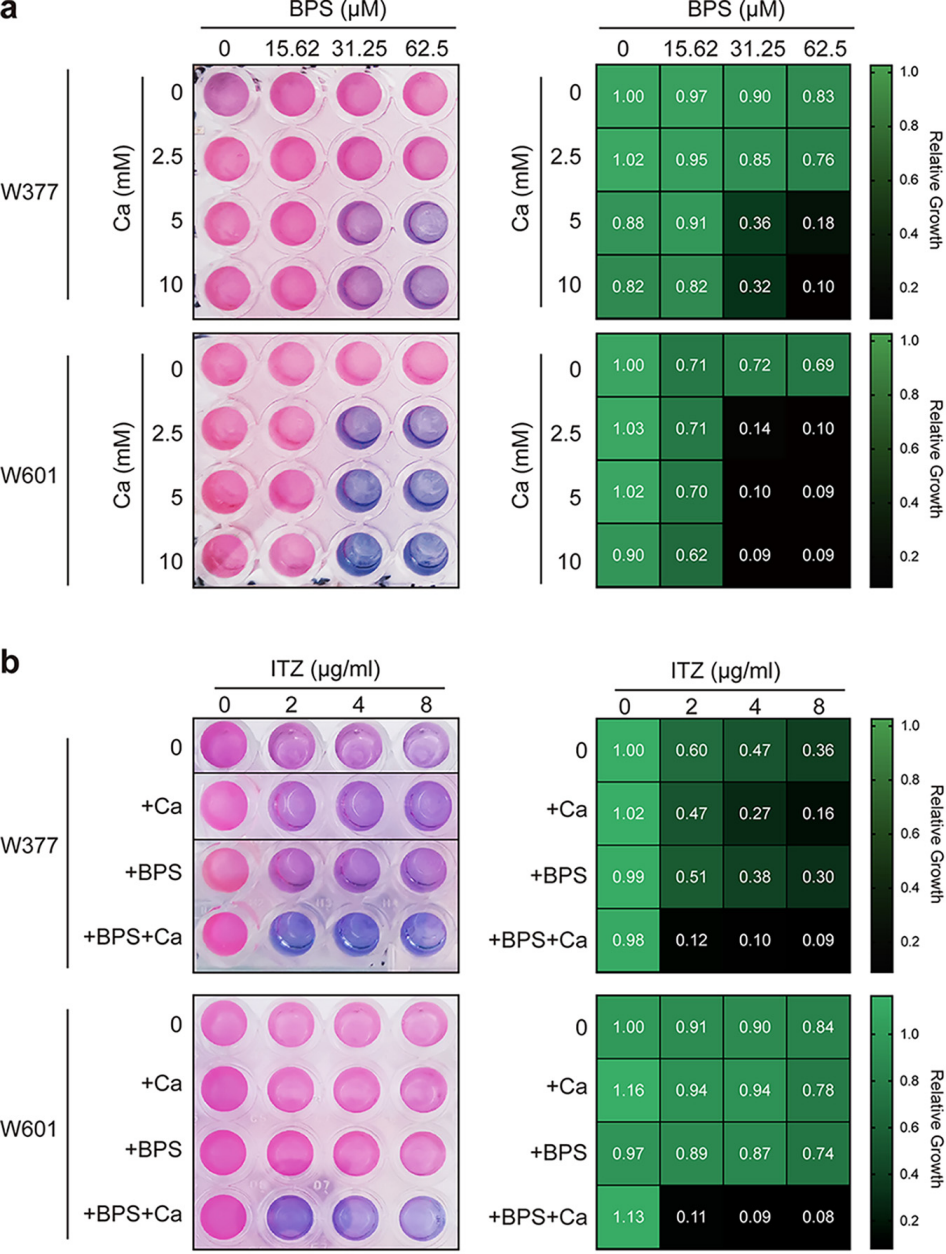
Supplementation of calcium with an iron chelator increases antifungal drug efficacy against azole-resistant A. fumigatus isolates. (a) Conidia (2 × 10^3^) of the A. fumigatus azole-resistant isolate W601, a laboratory-derived Cyp51A mutant, and W377, a non-Cyp51A mutant, were inoculated in 100 μL of MM−Fe with different concentrations of BPS in each column and supplemented 100 μl of MM-Fe with different concentrations of calcium for each row for incubation at 37°C. After 36 h, the medium in each well was replaced with 100 μL of medium supplemented with a final concentration of 0.002% (wt/vol) resazurin. The plate was then incubated for another 4 h at 37°C. Each well represents the mean from three replicate experiments. The heat maps show the growth ability of the wild-type with different concentrations of calcium under iron-deficient conditions. Assays were performed in triplicate, the optical density readings of fungal growth were standardized to the no-calcium-BPS control wells, and the results are shown as relative growth values. (b) Assays determining the susceptibility of W601 to the antifungal drug itraconazole combined with 50 μM BPS and 20 mM calcium and that of W377 to the antifungal drug itraconazole combined with 100 μM BPS and 2.5 mM calcium conidia (2 × 10_4_). After 48 h, the medium in each well was replaced with 100 μL of medium supplemented with a final concentration of 0.002% (wt/vol) resazurin. The plate was then incubated for another 13 h and 22 h at 37°C. Each well represents the mean from three replicate experiments. The heat maps show the growth ability of the wild-type with different concentrations of calcium under iron-deficient conditions. Assays were performed in triplicate, the optical density readings of fungal growth were standardized to the no-calcium-BPS control wells, and the results are shown as relative growth values.

Considering that the combination of calcium with an iron chelator inhibits the growth of the wild-type A. fumigatus, we wondered whether this also might have inhibitory effects on azole-resistant mutants. To test this, we employed the resazurin assay to assess the growth of the A. fumigatus azole-resistant isolate W601, which has a mutation in Cyp51A (azole drug target enzyme), and W377, which has a non-Cyp51A mutation, obtained from previous screening (66). As shown in Fig. 8a, similar to the inhibitory effects on the wild-type strain, supplementation of calcium with BPS significantly inhibited the growth of azole-resistant isolates (Fig. 8a). Similar effects were observed when a clinically approved iron chelator, deferoxamine (DFO), was used instead of BPS (Fig. S4). Moreover, we examined the effects of the combination of calcium with the iron chelator BPS on azole susceptibility with an *in vitro* assay. As shown in Fig. 8b, a resazurin assay showed that both azole-resistant isolates displayed hyperresistance to azoles. Notably, calcium supplementation with iron chelators dramatically enhanced the sensitivity to azoles. Collectively, these results suggest that cotreatment with calcium and iron chelators is an effective therapeutic option for the control of azole-resistant isolate growth.

## DISCUSSION

Iron is an essential trace element for both pathogens and their hosts. Within the past decade, much attention has focused on the roles of iron in animal virulence, as increasing evidence has suggested that iron is critically implicated in the host defense against invading pathogens (24). Calcium is a macronutritional component in all forms of life that is required for virtually all fundamental biological processes (33). In the human body, calcium is the most abundant metal (67). Accordingly, in fungi, calcium signaling also regulates morphogenesis, metabolism, the stress response, and virulence (57). However, although iron and calcium homeostasis has been extensively characterized, little is known about the relationship between iron and calcium. In the present study, we found that the addition of calcium interferes with iron absorption, especially in fungal pathogens in iron-deficient environments. Moreover, decreasing the iron availability in the medium enhances extracellular calcium influx intracellularly and in the mitochondria, suggesting that calcium could aggravate iron deficiency under iron-deficient culture conditions and that iron deficiency causes cellular abnormalities in calcium homeostasis.

It has been reported that fungal pathogens have evolved delicate systems to utilize nutrients important and essential for their survival from their surroundings or host niches (1, 2, 68). Under iron-deficient conditions, fungal pathogens can quickly sense the iron deficiency and activate reductive iron assimilation (RIA) and siderophore-mediated iron acquisition (SIA) to promote iron uptake. Interestingly, our findings indicate that iron deficiency, either in wild-type strains in iron-deficient media or in iron uptake-defective mutants, resulted in hypersensitivity to calcium in the three tested fungal species (Fig. 1b and d and 3). In addition, a mutant lacking *sreA* which displayed derepressed iron uptake that resulted in increased cellular accumulation of iron showed a slight increased resistance to calcium on harsh iron-deficient medium (Fig. S2), which is in contrast to results for A. fumigatus iron uptake-defective mutants. The addition of the iron chelator BPS exacerbated the calcium sensitivity phenotype in the three major classes of human pathogens tested.

These results suggest that growth inhibition under these conditions is not species specific. Notably, studies of human nutrients have shown that the concurrent ingestion of calcium and iron from the same meal inhibits iron absorption such that iron intake from a single meal was reduced regardless of whether the food sources contained calcium or calcium was supplemented (69). Moreover, Deshpande et al. indicated that calcium can negatively affect ferroportin activity (70). Gratz et al. demonstrated that in plants, iron deficiency induced cytosolic Ca^2+^ concentrations and the expression of calcineurin B-like proteins (71). Collectively, these phenomena suggest that calcium can inhibit iron absorption and that iron deficiency affects the calcium signaling response in many types of eukaryotic systems. However, there was no further mechanistic exploration regarding how calcium could affect iron absorption.

**FIG 9:**
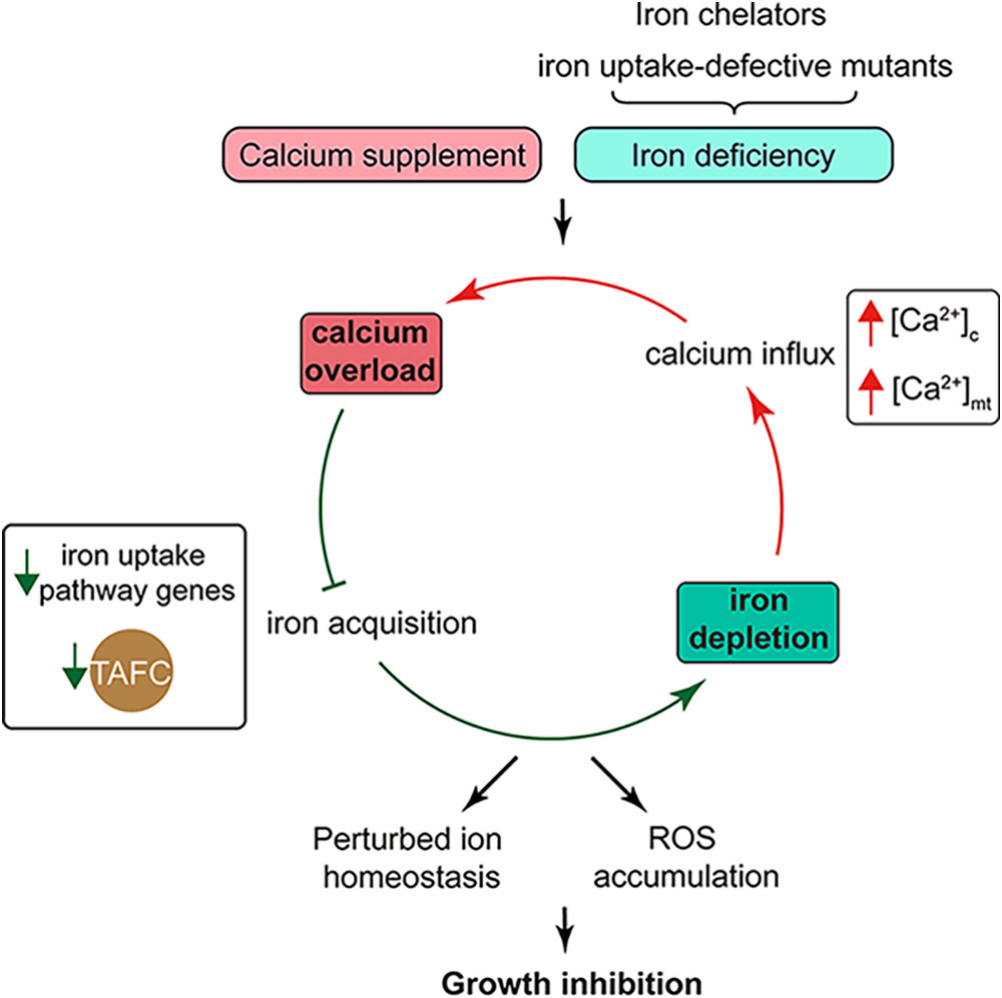
Model illustrating the mechanism by which a combination of iron deficiency and calcium excess inhibits fungal growth.

Here, we explored the possible molecular mechanism of interplay between calcium and iron as summarized in our model in Fig. 9. On the one hand, we found that calcium addition worsens iron absorption and completely inhibits cell growth. The possible reason was shown by qRT-PCR and Western blotting analyses (Fig. 4 and Fig. 7b and c). Calcium supplementation induced the downregulation of iron uptake-related genes, such as *sidA*, *sidJ*, and *freB*, and in particular caused a decrease in the expression of the transcription factor HapX, which tends to be highly expressed under iron-deficient conditions. Then, the decreased siderophore production of TAFC further aggravated the severe iron deficiency. On the other hand, iron deficiency using the iron chelator BPS and iron uptake-defective mutants showed hypersensitivity to calcium. Interestingly, deletion of the calcium channel CchA/MidA partially rescued the growth defects, indicating that excess calcium may enter cells from the predicted calcium channel. Low iron availability allows cells to mistakenly absorb calcium as a substitute, causing calcium abnormalities. Thus, iron-deficient conditions truly increased extracellular calcium influx and mitochondrial calcium accumulation, as shown in Fig. 5a and b and 6a.

Excitingly, based on these findings, we further verified that a combination of iron chelators or an iron-obtaining inhibitor along with calcium can serve as an efficient antifungal reagent to reduce the fungal burden *in vitro* and *in vivo*. Especially for azole drug-resistant A. fumigatus strains, combination treatment with the iron chelator and calcium is capable of inhibiting conidial germination and hyphal growth, as shown in Fig. 8a and Fig. S4. Thus, metal ion nutrients provide important avenues for the identification of novel antifungal candidates in both antifungal drug-susceptible and drug-resistant strains. However, further studies are required to investigate the clinical safety of using DFO, since the concentration of DFO used in this study was too high. On the other hand, DFO also supports iron supply of pathogens in mucormycosis *in vivo* (72, 73). Collectively, the findings in this study suggest that a microenvironment with excess calcium and limited iron is an efficient defense strategy to curb the growth of invading pathogens.

## MATERIALS AND METHODS

### Strains, media, and compounds.

The strains used in this study are summarized in Table S1. All chemicals used in this study were analytic reagents from the indicated companies or from Sigma-Aldrich Co. Generally, A. fumigatus strains were grown on minimal medium (MM) containing 1% (wt/vol) glucose and 70 mM NaNO_3_ as the sole carbon and nitrogen sources, respectively. MM+UU contained MM plus 5 mM uridine and 10 mM uracil. For iron-deficient conditions, iron was omitted from the trace element solution; for harsh iron-deficient conditions, the iron-specific chelator bathophenanthroline disulfonate (BPS) was added to the iron-deficient medium. Supplementation with calcium was carried out as described in the figure legends. Transformants were screened on medium containing 200 μg/mL hygromycin B (Sangon Biotech; A600230). The strains Cryptococcus neoformans H99 and Candida albicans ATCC 10231 were used for the experiments and maintained on yeast nitrogen base (YNB) medium (0.67% YNB medium, 2% dextrose) (74). Growth under low-iron conditions was performed in YNB with the addition of BPS (YNB-BPS). Supplementation with calcium was carried out or omitted, depending on the circumstances.

### Gene deletion and reconstitution.

All primers used in this study are listed in Table S2. For the generation of the *hapX* deletion cassette, as described previously (75), the fusion PCR technique was used. Briefly, approximately 1 kb of the upstream and downstream flanking sequences of the gene were amplified using the primer pairs P1/P3 and P4/P6, respectively. The gene *pyr4* from plasmid pAL5 was amplified with the primers *pyr4* F/R and used to restore *pyrG* function in the A1160 wild-type (WT) strain. Next, the three aforementioned PCR products were combined and used as templates to generate the gene deletion cassette using the primer pair P2/P5. This fragment was then used to transform the recipient strain A1160. Transformation of A. fumigatus was performed as described previously (55).

### Growth assays.

To assess the biomass of A. fumigatus on liquid media, the cells were incubated at 37°C for 24 h. For growth assays in liquid media with resazurin, A. fumigatus conidia (1 × 10^3^) were incubated at 37°C for 24 h. To analyze the phenotypes of C. neoformans and C. albicans in liquid media, fungal cells (1 × 10^5^) were grown in YNB, YNB+BPS, YNB+Ca, and YNB+BPS+Ca, and the flasks were incubated at 37°C. The optical density at 600 nm was measured every 4 h.

### Gene deletion and protein tagging.

To generate the GFP-labeled HapX strain, the 1.5-kb upstream sequence of *hapX*, referred to as fragment 1 (without the termination codon), and the downstream sequence of *hapX*, referred to as fragment 2 (including the termination codon), were amplified using hapX-GFP P1/P3 and hapX-GFP P4/P6, respectively. Fragment 3, containing a 5×GA linker, the enhanced GFP (eGFP) sequence, and the selection marker *hph*, used the primer pair GFP hph F/R. These three fragments were mixed and employed as a template to generate the *hapX*-GFP cassette using the fusion PCR technique with the primer pair hapX-GFP P2/P5. After purification of the *hapX*-GFP cassette, this fragment was used to transform the A1160 strain by homologous replacement of the original copy of the *hapX* gene to generate the *hapX*(p)::HapX-GFP strain, which had only one copy of *hapX*. Hoechst dye was used to label and visualize the cell nuclei of the GFP-labeled HapX strain. To constitutively express HapX with a FLAG tag, the ectopic integration method was used. Briefly, a 5×FLAG sequence was amplified using primers FLAG F/R from plasmid pFA6a-5×FLAGkanMX6, and the DNA sequence of HapX without a stop codon was amplified with HapX-FLAG F/R. These two fragments were then mixed and employed as a template to generate the HapX-FLAG cassette using the primer pair hapX-FLAG F and hapX-FLAG R. After purification of the PCR products, this fragment was used to transform strain A1160 by homologous replacement of the original copy of the *hapX* gene to generate the *hapX*(p)::HapX-FLAG strain, which had only one copy of *hapX*.

### Fluorescence microscopy analyses.

To visualize the localization of HapX-GFP under iron-deficient conditions in response to calcium, HapX-GFP strain was grown on coverslips in 1 mL of liquid minimal medium with or without iron at 37°C for 14 h before calcium stimulation. CaCl_2_ was added to a final concentration of 200 mM and incubated for 30 min. Cultured cells were then fixed with 4% paraformaldehyde and washed three times with phosphate-buffered saline (PBS). After that, the nuclear dye Hoechst dissolved in PBS was used at a final concentration of 1 μg/mL and incubated for 30 min at room temperature. The Hoechst solution was then removed, and the glass coverslip was washed three times with PBS. Images were captured using a Zeiss Axio imager A1 microscope (Zeiss, Jena, Germany) and arranged with Adobe Photoshop.

### Calcium content determination.

For calcium content determination, A. fumigatus conidia (1 × 10^7^ spores) were grown in parallel in iron-deficient media and minimal media (37°C, 24 h, 220 rpm) prior to the addition or not of 200 mM calcium for 60 min. Harvested mycelia were washed with double distilled water and shaken for 10 min with 0.1 M H_2_SO_4_ to eliminate calcium bound to the cell wall. The mycelia were dried in a drying oven at 60°C for 24 h and weighed. Fifty milligrams of freeze-dried mycelia was decomposed in closed polytetrafluoroethylene vessels containing 5 mL of HNO_3_ using a high-performance microwave digestion unit. Appropriate dilutions were made with distilled water, and the total calcium contents were determined by flame atomic absorption spectrometry.

### RNA-seq analysis, qRT-PCR analysis, and TAFC quantification.

Conidia (1 × 10^7^ spores) from the wild-type strain of A. fumigatus were inoculated in triplicate into liquid MM and iron-deficient medium and cultured for 24 h at 37°C prior to the addition (or not) of 200 mM CaCl_2_ for 10 min. The mycelia were then harvested and frozen in liquid nitrogen. For isolation of total RNA, mycelia were ground in liquid nitrogen. These RNA samples were prepared for digital transcriptome analyses by the RNA-seq approach. RNA-seq data are supplied in the supplemental material. For qRT-PCR analysis, total RNA was isolated from the frozen mycelium using kits (Sangon Biotech; B511361) as described in the manufacturer’s manual. The digestion of genomic DNA and synthesis of cDNA were performed using a HiScript II Q RT SuperMix for qPCR (+gDNA wiper) kit (Vazyme) as described by the supplier. The primers used for qRT-PCR analysis are listed in Table S2. To analyze the production of TAFC, the WT strains were cultured under iron-deficient conditions with or without the addition of calcium, and the supernatant was separated from the mycelia. Subsequently, the TAFC content of the supernatant was determined by reversed-phase HPLC as described previously (55).

### Cytoplasmic Ca^2+^ and mitochondrial Ca^2+^ transient measurements.

The WT strain, which carries the codon-optimized aequorin gene, was cultured in liquid MM in white 96-well plates at 37°C for 18 h away from light. The mycelia were washed with 200 μL of PGM (20 mM PIPES, 50 mM glucose, 1 mM MgCl_2_ [pH 6.7]) twice and then incubated in 100 μL of PGM containing a 2.5 μM concentration of the aequorin cofactor coelenterazine at 4°C for 4 h in the dark. After the samples were washed with PGM twice, the mycelia were incubated at room temperature for 1 h to recover their activity. Luminescence was recorded for a total period of 180 s. Calcium (100 mM) was added at 20 s, and then the active aequorin was completely discharged by the addition of 20% ethanol containing 2 M CaCl_2_ to calculate the total aequorin light emission of each well. The machine used in this study was an LB 96 Microplate luminometer. Finally, the relative luminescence units (RLU) were converted to [Ca^2+^]_c_ and [Ca^2+^]_mito_ using the following empirically derived calibration formula: pCa = 0.332588 (−log *k*) + 5.5593, where *k* is luminescence (in RLU) s^−1^/total luminescence (in RLU). Six parallel wells were used for each treatment. Each experiment was repeated at least three times.

### Reactive oxygen species measurements.

Briefly, A. fumigatus conidia were inoculated into liquid MM and MM−Fe media at 37°C, 220 rpm for 20 h. Then, CaCl_2_ was added to a final concentration of 0 mM and 200 mM, and the mixture was incubated for 4 h. DCFH-DA at a final concentration of 20 μM was added to the medium, which was then incubated at 37°C for 1.5 h. After that, the mycelia were harvested and washed with distilled water. The filtered mycelia were ground in liquid nitrogen and incubated in lysis buffer (50 mM HEPES, 1% Triton X-100 [pH 7.5]). The supernatant was collected by centrifugation at 15,000 × *g* and 4°C for 10 min. The fluorescence intensity was measured with an excitation wavelength of 504 nm and an emission wavelength of 524 nm. The relative content of ROS was normalized by the fluorescence intensity, subtracting the blank value from the total protein concentration measured by a Bio-Rad protein assay kit.

### Protein extraction and Western blotting.

For whole-cell lysate extraction, mycelia were ground with liquid nitrogen and the lysis buffer (0.2 M NaOH, 0.2% β-mercaptoethanol). Briefly, 20 mg of powdered mycelium was resuspended in 1 mL lysis buffer. Seventy-five microliters of 100% (wt/vol) trichloroacetic acid (TCA) was added, and the samples were vortexed and incubated on ice for 10 min. After centrifugation at 13,000 × *g* for 5 min at 4°C, the supernatants were removed and the pellets were heated to 95°C and vortexed in 100 μL of 1 M Tris and 100 μL of 2× SDS protein sample buffer until complete dissolution (Sigma). For analysis, FLAG and actin were detected with anti-FLAG mouse monoclonal antibody and antiactin antibody (ABclonal Technology Co.), respectively.

### Animal infection and histopathology.

Animal infection assays were performed in accordance with a method described previously with slight modifications (76, 77). Pathogen-free C57BL/6 mice were purchased from the Beijing Vital River Laboratory Animal Technology Co. A mouse model of iron deficiency with calcium supplementation was established in 8-week-old mice on a low-iron diet containing only 3 ppm iron (Trophic Animal Feed High-Tech Co., Ltd., China) and supplemented with 3% CaCl_2_; the iron concentration in a normal diet is 45 ppm. Mice were anesthetized by isoflurane inhalation, and then 5 × 10^7^ A. fumigatus conidia in 30 μL of PBS were instilled into the trachea by intratracheal instillation. At the indicated times, mice were killed, and lung tissues were isolated for detection of CFU and histopathological analysis. Briefly, for the immunosuppression model, C57BL/6 female mice were immunosuppressed on days −4 and −1 with 150 mg/kg cyclophosphamide. On day 0, the mice were infected with a suspension containing 2 × 10^6^ conidia or PBS as the control in a total volume of 30 μL via a tracheal cannula. To maintain immunosuppression, cyclophosphamide at 75 mg/kg of body weight was injected every 3 days after infection. To reduce animal distress, the body weights of the mice were recorded daily, and when the mice had lost 20% of their initial weight, they were euthanized. Mice were examined daily. Histological analysis of the lung tissue was carried out, and Grocott’s methenamine silver staining and HE staining were performed.

### Data availability.

The RNA-seq data have been deposited in the NCBI Sequence Read Archive under accession number PRJNA838924. Other relevant data supporting the findings of this study are available in this article and its supplemental material.
