# Supplementary material for: Synergistic Antifungal Effect of a Combination of Iron Deficiency and Calcium Supplementation
Source: Microbiol Spectr. 2022 Jun 8;10(3):e01121-22. doi: 10.1128/spectrum.01121-22 (PMC9241635; doi:10.1128/spectrum.01121-22)
Supplement: Supplemental file 1 — Supplemental material. Download spectrum.01121-22-s0001.pdf, PDF file, 0.7 MB [file spectrum.01121-22-s0001.pdf]

# Supplementary Information

## A synergistic antifungal effect by a combination of iron deficiency and calcium supplementation

**Authors:** Jing Ye, Yamei Wang, Xinyu Li, Qinyi Wan, Yuanwei Zhang*, Ling Lu*

Jiangsu Key Laboratory for Microbes and Functional Genomics, Jiangsu Engineering and Technology Research Centre for Microbiology; College of Life Sciences, Nanjing Normal University, Nanjing, China

*Address correspondence to Yuanwei Zhang, ywzhang@njnu.edu.cn; Ling Lu, linglu@njnu.edu.cn

**Supplementary Table 1.** *A. fumigatus* strains used in this study

| Strain name | Genotype | Reference |
|---|---|---|
| A1160 | Δ*aku80*; *pyrG1* | FGSC |
| WT | Δ*aku80*; *pyrG1*; *pyrG* | (1) |
| ATCC 10231 | | (2) |
| H99 | | (2) |
| Δ*leuB* | Δ*aku80*; *pyrG1*; Δ*leuB::pyr4* | (1) |
| Δ*hapX* | Δ*aku80*; *pyrG1*; Δ*hapX::pyr4* | This study |
| ΔmrsA | Δ*aku80*; *pyrG1*; Δ*mrsA::pyr4* | (3) |
| $WT^{AEQ}$ | Δ*aku80*; *pyrG1*; *AMA1::PgpdA::Aeq::pyr4* | (4) |
| Δ*leuB*$^{AEQ}$ | Δ*aku80*; *pyrG1*; Δ*leuB::hph*; *AMA1::PgpdA::Aeq::pyr4* | This study |
| Δ*hapX*$^{AEQ}$ | Δ*aku80*; *pyrG1*; Δ*hapX::hph*; *AMA1::PgpdA::Aeq::pyr4* | This study |
| $WT^{GMA}$ | Δ*aku80*; *pyrG1*; *AMA1::PgpdA::mt-Aeq::pyr4* | (4) |
| Δ*leuB*$^{GMA}$ | Δ*aku80*; *pyrG1*; Δ*leuB::hph*; *AMA1::PgpdA::mt-Aeq::pyr4* | This study |
| Δ*hapX*$^{GMA}$ | Δ*aku80*; *pyrG1*; Δ*leuB::hph*; *AMA1::PgpdA::mt-Aeq::pyr4* | This study |
| Δ*cchA* | Δ*aku80*; *pyrG1*; Δ*cchA::hph* | This study |
| Δ*midA* | Δ*aku80*; *pyrG1*; Δ*midA::pyr4* | This study |
| Δ*cchA*Δ*midA* | Δ*aku80*; *pyrG1*; Δ*cchA::hph*; Δ*midA::pyr4* | This study |
| $WT^{HapX\text{-}FLAG}$ | Δ*aku80*; *pyrG1*; *hapX::FLAG::hph* | This study |
| $WT^{HapX\text{-}GFP}$ | Δ*aku80*; *pyrG1*; *hapX::GFP::hph* | This study |
| W377 | | (5) |
| W601 | | (5) |

**Supplementary Table 2.** Primers used in this study

| Primer name | Sequence 5'-3' |
|---|---|
| For deletion | |
| pyr4 F | TGGCGTTACCCAACTTAATCG |
| pyr4 R | GCTTTCGGGAACTGGCTACTTAT |
| hph F | GAATTCCCTTGTATCTCTACACACAGGC |
| hph R | TCGAGTGGAGATGTGGAGTGGGCGCTTA |
| hph short F | CTTGAGCCTAAAATCCGCCG |
| hph short R | CGGAGCATTCACTAGGCAAC |
| leuB P1 | GTTCTACTTCAGCAGGTTTCGG |
| leuB P2 | AATACCTGATCCGGTCATAGCA |
| leuB P3 | GCCTGTGTGTAGAGATACAAGGGAATTCTTTTGAAGCCTACCATAGCCAC |
| leuB P4 | TAAGCGCCCACTCCACATCTCCACTCGAAAACTATCGCCTGAAGTACCTGG |
| leuB P5 | TGTAGGTCGCGTAGGTGTAGC |
| leuB P6 | TCATCTCCTGCCTCCCTAATC |
| hapX P1 | CTCAACCGAATGGTCAGCAA |
| hapX P2 | TGCTCATTATTCGCTCACCC |
| hapX pyr4 P3 | CGATTAAGTTGGGTAACGCCACAAGGGAACTGGAGCTAATG |
| hapX pyr4 P4 | ATAAGTAGCCAGTTCCCGAAAGCGCTTGTAGCATCACTCGCAT |
| hapX hph P3 | GCCTGTGTGTAGAGATACAAGGGAATTCGATTACGGAT GATGAGACT |
| hapX hph P4 | TAAGCGCCCACTCCACATCTCCACTCGATTTATCGCATC TCTGCTT |
| hapX P5 | CCATCTCTTCCAGGAAACGC |
| hapX P6 | TACACATCGCCCAGTATCCG |
| cchA P1 | CCCTTCACAACGCTACAAGACCT |
| cchA P2 | CGGAGATCGTAGAAGCAGCAATG |
| cchA hph short P3 | CGGCGGATTTTAGGCTCAAGGCCGAATGTAGACATTGCAGACT |
| cchA hph short P4 | GTTGCCTAGTGAATGCTCCGTTTTGTGCTTGGTGGGTGTTAGG |
| cchA P5 | TGAAGTAAGGTGCGGTTCGAGTG |
| cchA P6 | GGCTTGTACTCCTGCTTCGATTT |
| midA P1 | CAGATTTGAAGACAGCCGATAA |
| midA P2 | GACAAGACAGACGAACGCTTCA |
| midA P3 | CGATTAAGTTGGGTAACGCCAAACCAACTGGGCTCCGAAGGAC |
| midA P4 | ATAAGTAGCCAGTTCCCGAAAGCGAACCAGGTTCCAATGATACCC |
| midA P5 | GCACGAGAATACCAGGCTACCG |
| midA P6 | CAAATACACCAGCGAAGACAAT |
| For gene label | |
| hapX GFP P1 | ACCAAAACCAGGCAGGAAA |
| hapX GFP P2 | AGGTATCGGTGGAAAGAAG |
| hapX GFP P3 | CCAGCGCCTGCACCAGCTCCTTTGTCGGCAAACCGTCGGTC |
| hapX GFP P4 | CACTCCACATCTCCACTCGATGATTTATCGCATCTCTGCTTG |
| hapX GFP P5 | GAAGTGATGGTTAGTGGTG |
| hapX GFP P6 | CTTTTCTGGGGTCTGGTCT |
| GFP hph F | GGAGCTGGTGCAGGCGCTGG |

| | |
|---|---|
| GFP hph R | TCGAGTGGAGATGTGGAGTG |
| FLAG F | CTCGAGGGATCCCCGGGAATG |
| FLAG R | GACATTCCTTTTACCCGGGCTA |
| hapX FLAG F | TCCAGGACTGATAACCACG |
| hapX FLAG R | CATTCCCGGGGATCCCTCGAGTTTGTCGGCAAACCGT CGGTC |
| For RT-PCR | |
| RT pmcA F | CGTCGGTGCTGCCAACGATT |
| RT pmcA R | GACACCGTCGACTGGGACGA |
| RT pmcB F | GAACCGCCAAGCGACTCCAC |
| RT pmcB R | GCAGCATTGCCACCTCGGTT |
| RT pmcC F | TTGGGAACAGGGCACCGACT |
| RT pmcC R | TCCGCAACGAGGCTCGAGAA |
| RT cchA F | GGGATCCAGAGCTTCAAGTC |
| RT cchA R | TGCAGGATGTTATCGAAATTG |
| RT midA F | GCCGTTCACAATGTTTGCTCAT |
| RT midA R | AACTGCTCCTTCGGTTTCTTGC |
| RT mcuA F | CCTGGACAGGGCGACAAGGA |
| RT mcuA R | TCGAATGACGGCACGGCAAC |
| RT freB F | GCAAACTCGCACTGCGTTTCTG |
| RT freB R | CTGCTCGCCGTATGGTCCTTCA |
| RT hapX F | CCGCACCATCCTTGACTTTAT |
| RT hapX R | CGAGGTTTAGGCAAGGTATGAA |
| RT mirB F | AAGCCGAGAAAAAGGGGG |
| RT mirB R | AACCCAGATGAAGCCCAG |
| RT sidF F | CCAGTTATGCGATCCATGTTGT |
| RT sidF R | TCAAAGGCAATGTCGGTGTAGG |
| RT sidG F | CAAAAGCCCAGCATGACTC |
| RT sidG R | CCTACGCCCTTGGAAAATA |
| RT sidH F | TGACGATGGAAGGGCTGCATTA |
| RT sidH R | TGGCTTTCGCTTCTCCACAAAT |
| RT sidJ F | GACCTCCTCGTACCAATCCTGG |
| RT sidJ R | GTGAAGCACGCACTGGCTACCT |
| RT tub F | TTCCGTCCCGACAACTTCGT |
| RT tub R | CACAGCCTTCAGCCTCACG |

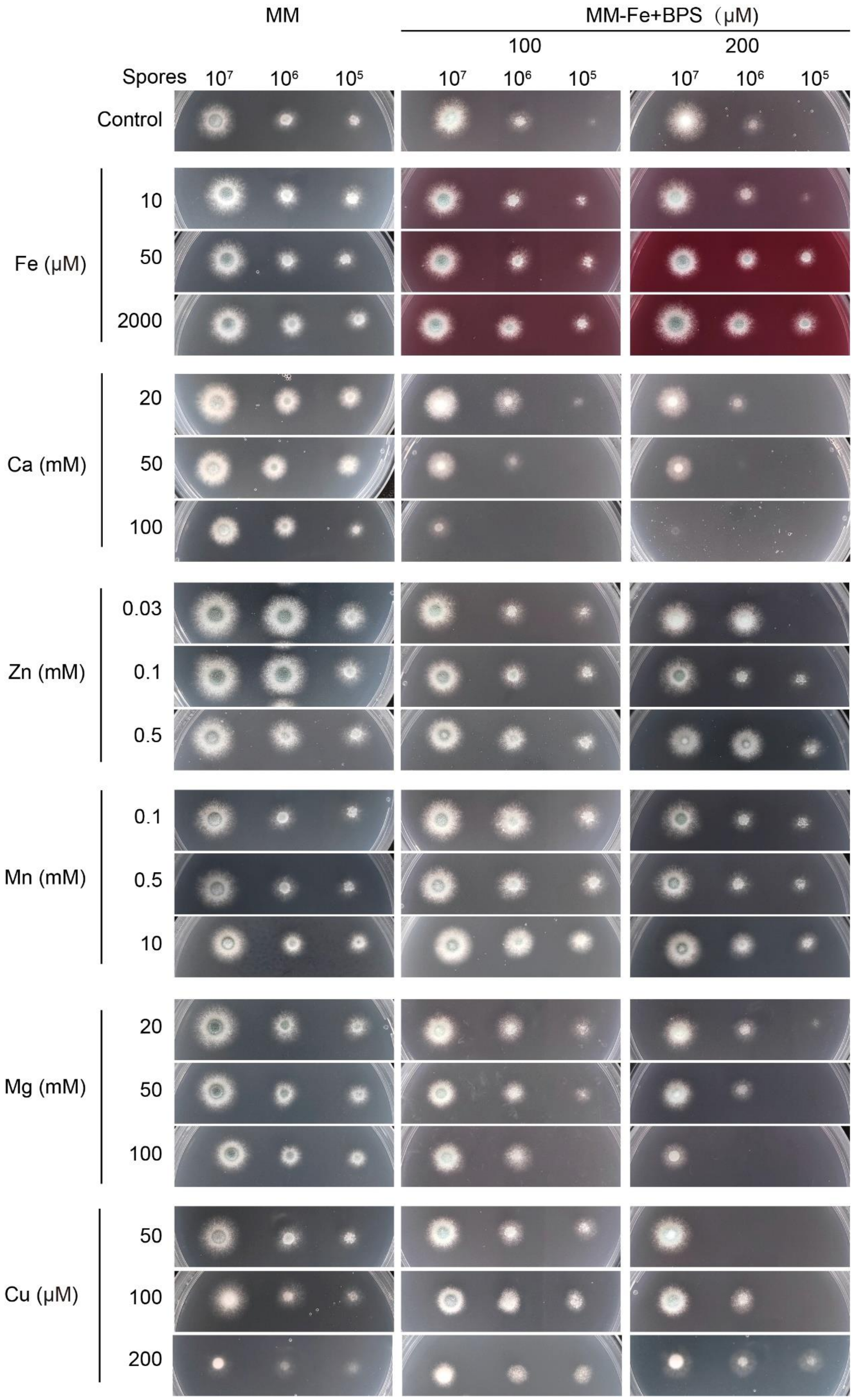
MM
MM-Fe+BPS（μM)
100
200
Spores
$10^7$
$10^6$
$10^5$
Control
Fe (μM)
10
50
2000
Ca (mM)
20
50
100
Zn (mM)
0.03
0.1
0.5
Mn (mM)
0.1
0.5
10
Mg (mM)
20
50
100
Cu (μM)
50
100
200

**Supplementary Figure 1: Calcium affect fungal growth under iron-deficient conditions non-specifically.** Growth phenotype of the wild-type strain on solid minimal medium MM and harsh iron-deficient medium MM-Fe+BPS with various divalent ions in different concentrations at 37°C for 40 hours. Data represent the means of biological replicates.

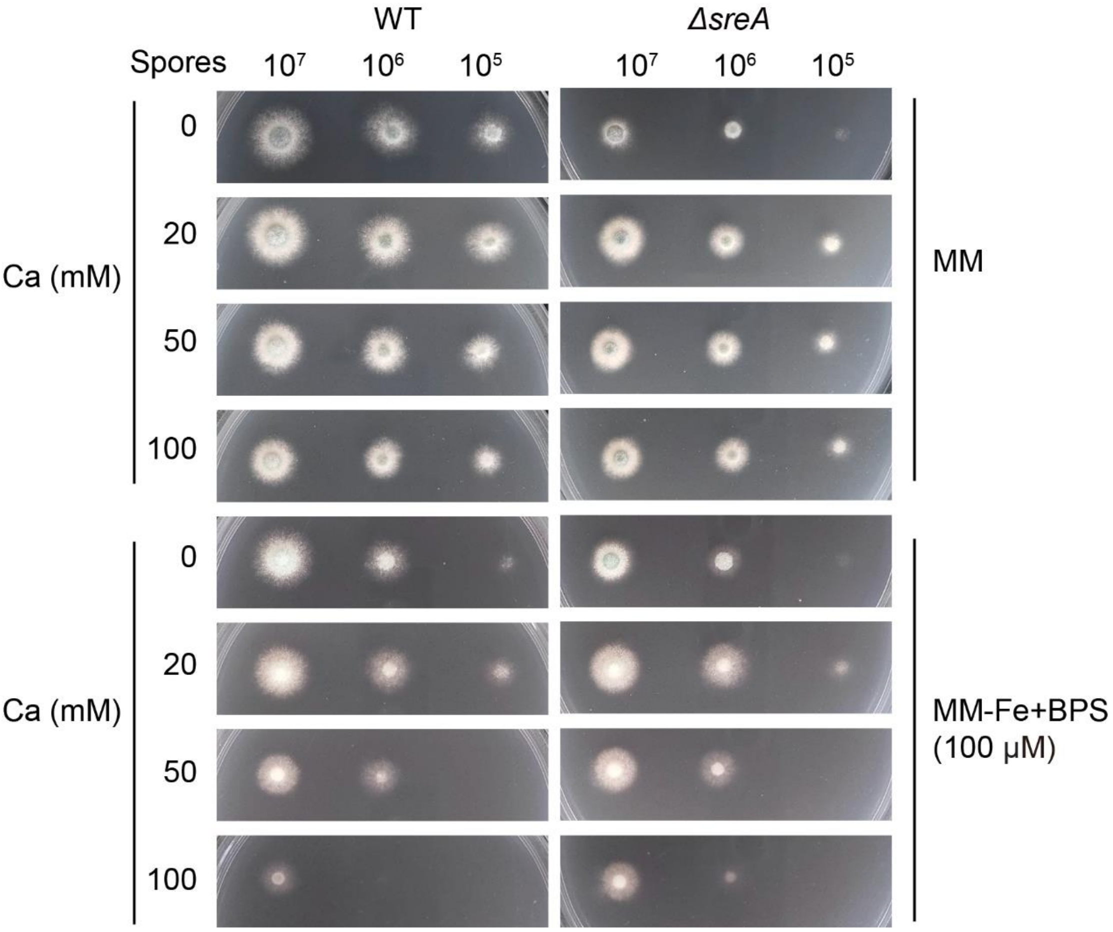


**Supplementary Figure 2: *A. fumigatus sreA* mutants show increased resistance to calcium under iron-deficient conditions.** Growth phenotypes of the wild-type 1160[c] (WT) and Δ*sreA* strains grown on solid minimal medium MM and harsh iron-deficient medium MM-Fe+BPS supplemented with calcium in different concentrations.

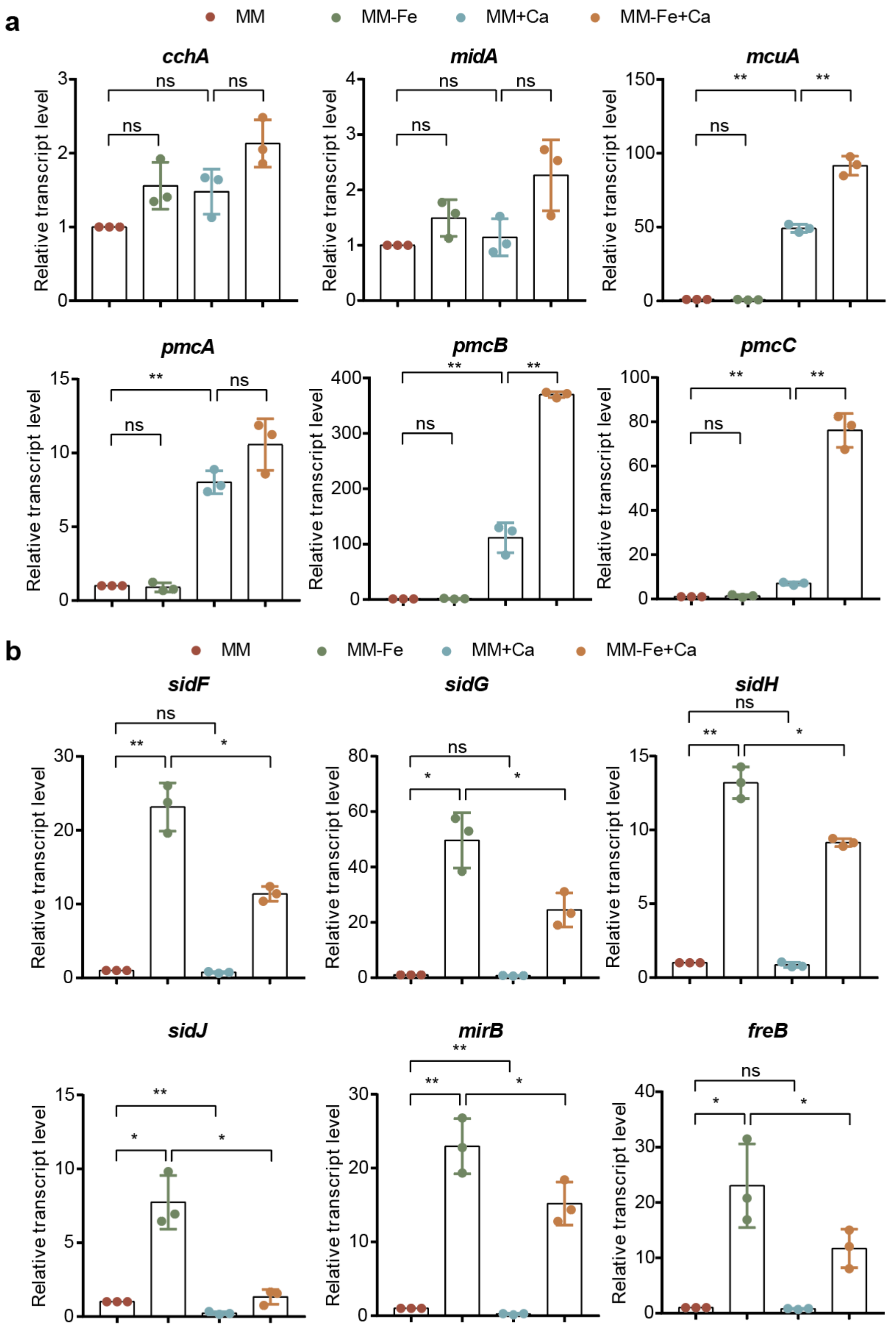


**Supplementary Figure 3: The transcript level verification by qRT-PCR for the selected genes from RNA-seq.** The wild-type strain was grown for 24 h at 37˚C and transferred to 200 mM $CaCl_2$ for 0 and 10 mins in MM and MM-Fe, respectively. (A) Verification for genes involved in calcium homeostasis. (B) Verification for genes involved

in iron uptake. Standard deviations present the average of three independent biological repetitions. Statistical significance was determined using a two-tailed t test, ns, not significant; $*P < 0.05$; $**P < 0.01$.

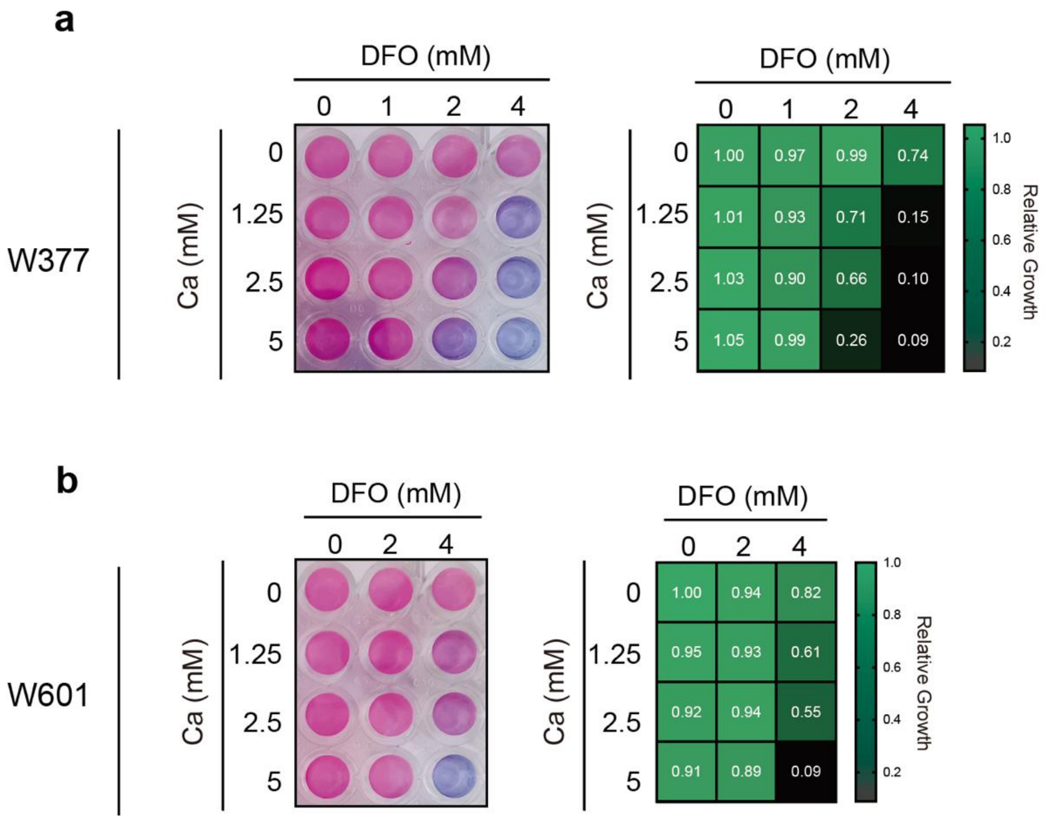


**Supplementary Figure 4: Supplementation of calcium with an iron chelator DFO inhibits growth of azole-resistant *A. fumigatus* isolates.** Conidia (2 x $10^4$) of the *A. fumigatus* azole-resistant isolate W601 (a), a laboratory-derived Cyp51A mutant, and W377 (b), a non-Cyp51A mutant, were inoculated in 100 µl of MM-Fe with different concentrations of DFO in each column and supplemented with different concentrations of calcium for each row for incubation at 37 °C. After 36 h, the medium in each well was replaced with 100 µl of medium supplemented with a final concentration of 0.002% (w/v) resazurin. The plate was then incubated for another 4 h at 37 °C. Each well represents the mean of three replicate experiments. The heatmaps show the growth ability of the wild-type with different concentrations of calcium under iron-deficient conditions. Assays were performed in triplicate, the optical density readings of fungal growth were standardized to the no-calcium-DFO control wells, and the results are shown as relative growth values.

**Reference**


1 Long, N. et al. The Zn2Cys6-type transcription factor LeuB cross-links regulation of leucine biosynthesis and iron acquisition in *Aspergillus fumigatus*. *PLoS Genet* 14, e1007762, (2018).

2 Li, X., Li, Y., Wang, R., Wang, Q. & Lu, L. Toxoflavin Produced by *Burkholderia gladioli* from *Lycoris aurea* Is a New Broad-Spectrum Fungicide. *Appl Environ Microbiol* 85, (2019).

3 Long, N., Xu, X., Qian, H., Zhang, S. & Lu, L. A Putative Mitochondrial Iron Transporter MrsA in *Aspergillus fumigatus* Plays Important Roles in Azole-, Oxidative Stress Responses and Virulence. *Frontiers in Microbiology* 7, (2016).

4 Li, Y. et al. Mitochondrial dysfunctions trigger the calcium signaling-dependent fungal multidrug resistance. *Proc Natl Acad Sci U S A* 117, 1711-1721, (2020).

5 Wei, X. et al. Screening and Characterization of a Non-cyp51A Mutation in an *Aspergillus fumigatus* cox10 Strain Conferring Azole Resistance. *Antimicrob Agents Chemother* 61, (2017).
